# The Effects of Microbiome Modulating Therapies on Inflammatory Markers in Autoimmune Disease: A Systematic Review and Meta-Analysis

**DOI:** 10.3390/nu18040560

**Published:** 2026-02-08

**Authors:** Ghalya Ashkanani, Mlaak Rob, Mahmoud Yousef, Alia Ashkanani, Yousef A. Al-Najjar, Sa’ad Laws, Ali Chaari

**Affiliations:** Weill Cornell Medical College Qatar, Qatar Foundation, Education City, Doha P.O. Box 24144, Qatar; gsa2002@qatar-med.cornell.edu (G.A.); mtr4001@qatar-med.cornell.edu (M.R.); mmy4002@qatar-med.cornell.edu (M.Y.); asa2028@qatar-med.cornell.edu (A.A.); yaa4004@qatar-med.cornell.edu (Y.A.A.-N.); sal2018@qatar-med.cornell.edu (S.L.)

**Keywords:** autoimmune diseases, gut microbiota, probiotics, synbiotics, fecal microbiota transplant, systematic review

## Abstract

Background: Autoimmune diseases (ADs) are a growing global health burden, driven by chronic inflammation and immune dysregulation. The gut-immune axis plays a central role in their pathogenesis, with dysbiosis linked to several conditions. This has prompted investigation into nutraceuticals such as probiotics, prebiotics, synbiotics, and fecal microbiota transplantation as adjunctive therapies. Methods: We conducted a systematic review and meta-analysis following PRISMA guidelines, searching PubMed, Embase, and Web of Science for randomized controlled trials evaluating these interventions in autoimmune diseases. Results: Twenty-eight randomized control trials (RCTs) involving 2002 patients across 11 countries met inclusion criteria. Across the included RCTs, pooled analyses demonstrated significant reductions in c-reactive protein (CRP) (SMD −0.67, 95% CI −1.00 to −0.33; I^2^ = 80.8%) and Tumor necrosis factor-alpha (TNF-α) (SMD −1.81, 95% CI −2.67 to −0.94; I^2^ = 96%), a significant increase in Interleukin-10 (IL-10) (SMD 2.65, 95% CI 0.64 to 4.66; I^2^ = 98%), and no overall significant effect on Interleukin-6 (IL-6) (SMD −0.89, 95% CI −1.99 to 0.22; *p* = 0.12). The strongest evidence of benefit was observed in rheumatoid arthritis, multiple sclerosis, and inflammatory bowel disease. Pooled effects are limited by extreme between-study heterogeneity (I^2^ 80–98%), leaving interpretation as exploratory rather than definitive. More limited or inconsistent findings were reported for systemic lupus erythematosus, hypothyroidism, axial spondylarthritis, and juvenile idiopathic arthritis. Heterogeneity in study design, probiotic strain selection, dosage, and treatment duration limited comparability across trials. Conclusions: Overall, microbiome-targeted nutraceuticals appear promising for attenuating systemic inflammation in select autoimmune conditions, but results remain mixed. Larger, rigorously designed RCTs with standardized endpoints are needed to clarify efficacy, identify optimal formulations, and define patient populations most likely to benefit.

## 1. Introduction

Autoimmune diseases (ADs) are a group of complex and diverse disorders in which the body’s immune system attacks its host cells, leading to a state of chronic inflammation [1]. They arise from the human body’s failure to recognize self from non-self, a phenomenon known as loss of immune tolerance [2]. As a result, ADs significantly affect quality of life, with patients experiencing a wide range of physical symptoms and psychological distress [3].

In the past, prevalence of ADs was low; however, incidence is increasing due to advancements in diagnostics and increased awareness in the general population [4]. Recent studies have established that ADs affect one in ten individuals, though rates of incidence and prevalence vary depending on types of ADs and geographical locations [5]. For example, a cross-sectional study in Qatar recognized rheumatoid arthritis (RA), connective tissue disease, and inflammatory bowel disease (IBD)-associated arthritis as the most common autoimmune inflammatory disorders [6,7]. Globally, prevalence estimates for ADs differ due to variations in study methodologies, population characteristics, and diagnostic criteria. Hypothyroidism is among the most prevalent ADs, affecting approximately 2% of the global population, closely followed by RA with a similar prevalence [8]. In contrast, diseases like type 1 diabetes mellitus (T1DM), Crohn’s disease (CD), and psoriasis demonstrate moderate prevalence rates, generally below 1% [9,10,11]. Meanwhile, multiple sclerosis (MS) and ulcerative colitis (UC) are among the least common, with each affecting less than 1% of the population worldwide [12]. These differences highlight the complexity of assessing the global burden of ADs.

Beyond their epidemiology, ADs develop through a complex interplay of genetic, environmental, and immunological factors that disrupt immune tolerance. Genetic susceptibility is well-documented, with twin studies demonstrating higher concordance rates for ADs in monozygotic twins compared to dizygotic twins [13]. T1DM and RA exemplify this genetic influence, showing significant familial concordance often involving multiple genes [14]. Environmental factors also play a crucial role in AD development, as seen in conditions like celiac disease and drug-induced lupus, where external factors initiate immune dysregulation [13]. In this context, there are over 100 differentiated ADs, with some affecting multiple organs (e.g., systemic lupus erythematosus (SLE)) and others targeting a single organ (e.g., primary biliary cholangitis (PBC)).

Building on this pathophysiological framework, emerging evidence shows the gut microbiota as a critical factor in AD pathogenesis [15]. The gut microbiota is a complex community of microorganisms crucial for immune regulation, metabolism, synthesis, and maintaining gut barrier integrity [16]. Disruptions in this intricate balance, known as dysbiosis, can lead to inflammation, immune dysfunction, and contribute to the onset and progression of ADs by promoting a leaky gut and triggering autoreactive immune responses [17,18]. ADs including RA, IBD, MS, SLE, T1DM, and psoriasis have all been associated with alterations in composition and homeostasis of the gut microbiota, with many diseases such as RA demonstrating variations in microbiota composition across disease stages [19,20,21]. These microbial dysregulations promote ADs by a range of mechanisms including immune cell translocation, elevation of reactive oxygen species (ROS), and upregulation of proinflammatory cytokines and chemokines, including interleukin-12 (IL-12), interleukin-23 (IL-23), and type I interferons [21,22].

Despite progress in understanding these mechanisms, conventional treatments remain limited. Corticosteroids are still a cornerstone of therapy, but the severity of its side effect profile creates a barrier to usage. Some of the known side effects of corticosteroids include hypertension, metabolic syndrome, bone fractures, and cataracts [23]. Given these limitations, microbiome-targeted interventions, such as probiotics, prebiotics, synbiotics, and fecal microbiota transplantation (FMT), are being explored for their potential to restore microbial balance and improve clinical outcomes in ADs [24,25,26,27]. These therapies promote beneficial microbes, suppress pathogenic strains, and modulate immune function, showing promise in clinical and experimental studies [28,29,30,31].

These findings suggest that microbiome-modulating therapies could play a role in reducing inflammation and disease severity in ADs. Accordingly, this paper aims to systematically review and meta analyze the evidence on the effects of gut microbiome-targeted nutraceuticals, including probiotics, prebiotics, synbiotics, and FMT on inflammation and oxidative stress in ADs. Specifically, we aim to examine their impact on inflammatory markers such as c-reactive protein (CRP), IL-6, IL-10, and Tumor necrosis alpha (TNF-α) in a meta-analysis, with other markers qualitatively noted as well.

## 2. Methods

### 2.1. Study Protocol and Literature Search

This systematic review followed the guidelines outlined in the Preferred Reporting Items for Systematic Reviews and Meta-Analyses (PRISMA) statement [32]. The literature search was conducted across PubMed (National Library of Medicine, Bethesda, MD, USA), Web of Science Core Collection (Clarivate Analytics, Philadelphia, PA, USA), and Embase (Elsevier, Amsterdam, The Netherlands) on 8 September 2024 without restrictions on language or year of publication. Detailed search strategies for each database are provided in the Supplementary Materials. Additional searches of the gray literature were conducted through reference lists, author publications, review articles, conference abstracts, relevant websites, and ClinicalTrials.gov to identify unpublished studies. A recursive search was further carried out by examining the bibliographies of all retrieved articles.

### 2.2. Inclusion and Exclusion Criteria

This review included randomized clinical trials with control or placebo groups that examined the effects of probiotics (bacteria and yeast-based), prebiotics, synbiotic, or FMT administration on inflammatory and oxidative stress markers (e.g., ESR, CRP, IL-6, TNF-α) in patients with ADs. Studies were eligible regardless of follow-up duration, participant demographics (age, sex, ethnicity), geographic location, or publication year. Studies were excluded if they were non-clinical, such as reviews, books, editorials, letters, surveys, guidelines, conference proceedings, abstracts, or animal studies. Additional exclusion criteria included studies that did not report on inflammatory or oxidative stress markers, focused on conditions other than ADs, or were published in languages other than English, or were duplicates.

### 2.3. Outcome Measures

As no consensus exists on a standardized set of biomarkers for evaluating treatment effects in autoimmune diseases, we included the most frequently reported outcomes across eligible trials. The primary outcomes assessed were inflammatory markers measured before and after intervention, specifically CRP, IL-6, TNF-α, and IL-10. These variables were extracted and analyzed as continuous outcomes, representing the change from baseline to the end of intervention for both probiotic and control groups. Additional biomarkers, including both inflammatory and oxidative, such as erythrocyte sedimentation rate (ESR), glutathione (GSH), malondialdehyde (MDA), and total antioxidant capacity (TAC) were qualitatively reviewed but not included in the quantitative meta-analysis due to insufficient comparable data across studies.

### 2.4. Screening and Data Extraction

The search results from each database were merged and imported into Covidence, an online tool for systematic review management (Veritas Health Innovation, Melbourne, VIC, Australia; version 2). The title, abstract, and full text were screened by two independent authors. Next, data were systematically collected and organized into a comprehensive table using Microsoft Excel Sheets (Microsoft Corporation, Redmond, WA, USA; Microsoft Excel for Microsoft 365, version 16.78). For each study, essential information was extracted, including study identification details (author, year, country, study type, etc.), intervention specifics (type, dosage, route, and duration), placebo and control group demographics, and the studied autoimmune disease. Outcome measures were extracted in means ± standard deviations format at baseline and end of intervention with their respective changes for both intervention and placebo groups.

For quantitative synthesis, change-from-baseline mean ± SD values were preferentially extracted; when unavailable, endpoint values were used instead in accordance with Cochrane Handbook guidance, without imputing baseline-follow-up correlations. Studies reporting outcomes only as medians with interquartile ranges or lacking sufficient data for variance estimation were excluded from meta-analysis and summarized narratively, and no median-to-mean transformations were performed.

### 2.5. Risk of Bias Assessment

Risk of bias was assessed using the Cochrane Risk of Bias 2 (RoB 2) tool. This tool examines 5 domains: D1 (randomization), D2 (adherence to interventions), D3 (missing data), D4 (outcome measurement), and D5 (reporting). Each item was rated low risk, some concern, or high risk of bias. The overall risk of bias was determined based on the score of the lowest category of each respective study.

### 2.6. Statistical Methods

All statistical analyses and generation of forest plots were conducted using Stata software version 16 (StataCorp, College Station, TX, USA, version 16) with dedicated meta-analysis modules. For each outcome, standardized mean differences (SMD) with corresponding 95% confidence intervals were calculated using pooled data from randomized controlled trials evaluating similar outcome. Random-effects models were applied throughout to account for expected heterogeneity among studies in design, population, dosage, and duration. Between-study heterogeneity was quantified using the I^2^ statistic and Q statistic. Potential publication bias was examined through visual inspection of funnel plots and formal testing with Egger’s regression method. Prespecified subgroup analyses were performed based on intervention type (probiotic, synbiotic, or fecal microbiota transplantation), duration of intervention (≤8 weeks vs. >8 weeks), dosage (low (<1 × 10^10^) vs. high (≥1 × 10^10^)), and by AD. Publication bias was assessed visually using a funnel plot and statistically using Egger’s regression test and Begg’s rank correlation test. Funnel plot asymmetry was evaluated by plotting effect sizes against their standard errors, with contour-enhanced regions representing 90%, 95%, and 99% confidence intervals.

## 3. Results

### 3.1. Study Selection

**Figure 1:**
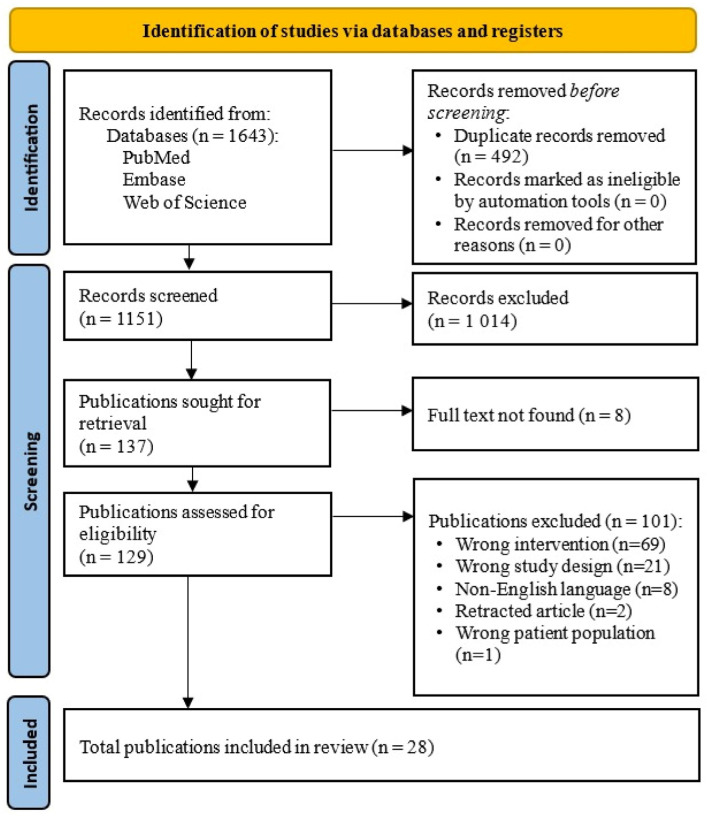
PRISMA flowchart depicting the search strategy and study selection process.

A comprehensive literature search initially identified 1643 studies. Following the removal of 492 duplicates (10 manually and 482 by Covidence), 1151 unique studies remained for screening. Of these, 1014 studies were excluded based on initial relevance and inclusion criteria. Next, we sought to retrieve the full text of 137 publications, and 129 studies underwent full-text assessment for eligibility. During this phase, 101 studies were excluded for reasons including wrong intervention (69), wrong study design (21), non-English language (8), retraction (2), and irrelevant patient populations (1). Ultimately, 28 studies met all eligibility criteria and were included in this systematic review (Figure 1).

### 3.2. Risk of Bias and Study Characteristics

**Figure 2:**
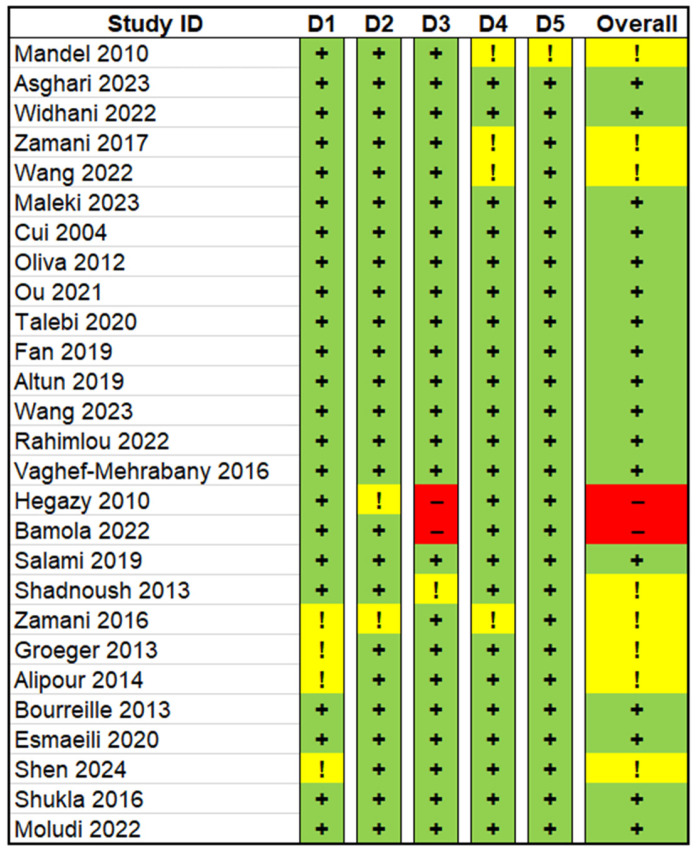
Schematic presentation of bias risk assessment using Cochrane Rob2 tool. Green cells with a “+” indicate low risk of bias, yellow cells with an “!” indicate some concerns, and red cells with a “–” indicate high risk of bias [33,34,35,36,37,38,39,40,41,42,43,44,45,46,47,48,49,50,51,52,53,54,55,56,57,58,59].

Eighteen studies were determined to have low risk of bias. Eight studies had some concerns in at least one domain, and two studies had a high risk of bias (Figure 2). Four papers had bias in D1 (randomization process), two had bias in D2 (deviation from intended intervention), three had bias in D3 (missing outcome data), four had bias in D4 (measurement of outcome), and one had bias in D5 (selection of reported result). In terms of publication bias, visual inspection of the funnel plot revealed a relatively symmetric distribution of studies around the pooled effect estimate (Supplementary Figure S1). Egger’s regression test (t = −1.17, df = 14, *p* = 0.26) and Begg’s rank correlation test (z = −1.17, *p* = 0.24) indicated no significant publication bias. Publication bias was assessed using visual inspection of funnel plots and Egger’s regression test and Begg’s rank correlation test only for outcomes with 10 studies or more, in accordance with established methodological recommendations. Accordingly, formal assessment of publication bias was performed for CRP (16 studies). For other cytokines, the number of included studies was insufficient to allow meaningful funnel plot asymmetry testing, and publication bias was therefore not formally evaluated for these outcomes.

In total, this study included 2002 patients, comprising 993 in the intervention group and 1009 in the placebo group. The studies were conducted across 11 countries, with the largest number conducted in Iran (12 studies), followed by China (5 studies), and India (3 studies), with one study being conducted in each of Egypt, France, Indonesia, Ireland, Italy, Taiwan, Turkey, and USA. The studies were published between 2004 and 2024, with the highest number in 2022 (13 studies). The median publication year was 2019 (interquartile range (IQR) 2015–2022). Study characteristics can be found in Table 1.

Study duration ranged from 1 to 52 weeks, with a median of 8 weeks (IQR: 8–12 weeks). Oral administration was the predominant delivery method, used in 93.5% of studies (29 studies). The interventions primarily included probiotic supplements (20 studies), followed by synbiotic supplements (6 studies), probiotic enema (1 study), and FMT (1 study). Of the 28 studies, 14 (50%) used multi-strain probiotics, 8 (29%) used single-strain probiotics, and all synbiotic studies (21%) used multi-strain formulations. From the trials that provided the dosages, probiotic-only studies delivered a median dose of 2 × 10^9^ colony forming unit (CFU)/day (range: 1 × 10^8^ to 112.4 × 10^9^ CFU/day, IQR: 1.6 × 10^9^–6.4 × 10^9^ CFU/day). Trials using synbiotics administered a median daily dose of 4.5 × 10^9^ CFU and prebiotics a median dose of 475 mg/day, typically using fructooligosaccharides or inulin as the prebiotic component. The intervention group had a mean age of 38.97 years and a mean Body Mass Index (BMI) of 26.05, while the placebo group had a mean age of 39.30 years and a mean BMI of 25.

**Table 1:** Summary of randomized, placebo-controlled clinical trials evaluating the effects of probiotic and synbiotic interventions on inflammatory and oxidative stress biomarkers in patients with ADs. Details include author, country, type of study, disease, intervention type, dose, duration, sample sizes, biomarker changes in placebo versus intervention groups, direction of effect, and statistical significance between groups. Bolded is significant values.

Author	Country	Type of Study	Disease	Intervention Type	Intervention, Dose, Duration	Intervention Group Participants	Control Group Participants	Inflammatory/Oxidative Marker	Intervention Effect	Placebo Change	Intervention Change	Statistical Significance Between Groups
Mandel 2010 [33]	USA	Randomized, double-blind, placebo-controlled, parallel-design, clinical pilot trial	RA	Probiotics	*Bacillus coagulans* GBI-30, 6086, 2 billion CFU per day, with one caplet daily, 60 days	22	22	CRP	No change	Not reported	0.008 ± 0.78	*p* = 0.98
								ESR	Increased	Not reported	0.054 ± 0.64	*p* = 0.8
Vaghef-Mehrabany 2016 [34]	Iran	Randomized, double-blind, placebo-controlled clinical trial	RA	Probiotics	Daily probiotic capsule containing *Lactobacillus casei* 01 at 10^8^ CFU, 8 weeks	22	24	SOD	Decreased	16.81 ± 193.84 *p* = 0.547	65.91 ± 202.97 *p* = 0.003	*p* = 0.2
								GPx	Decreased	0.97 ± 0.97 *p* = 0.032	1.30 ± 3.27 *p* = 0.001	*p* = 0.477
								CAT	Decreased	7.55 ± 22.51 *p* = 0.116	9.00 ± 20.57 *p* = 0.189	*p* = 0.762
								TAC	Increased	0.02 ± 0.18 *p* = 0.35	0.02 ± 0.17 *p* = 0.401	*p* = 0.359
								MDA	Decreased	0.37 ± 1.57 *p* = 0.088	0.28 ± 1.05 *p* = 0.212	*p* = 0.445
Zamani 2016 [35]	Iran	Randomized, double-blind, placebo-controlled clinical trial	RA	Probiotics	Daily probiotic capsule containing *Lactobacillus acidophilus* (2 × 10^9^ CFU/g), *Lactobacillus casei* (2 × 10^9^ CFU/g), and *Bifidobacterium bifidum* (2 × 10^9^ CFU/g), 8 weeks	30	30	**CRP**	**Decreased**	**3.07 ± 5.53** ***p* = 0.001**	**0.66 ± 2.56** ***p* = 0.25**	***p* < 0.001**
								NO	Decreased	2.8 4.3 *p* = 0.001	0.9 ± 7.2 *p* = 0.58	*p* = 0.12
								TAC	Increased	24.4 ± 198.6 *p* = 0.5	17.9 ± 171.5 *p* = 0.57	*p* = 0.889
								MDA	No change	0.2 ± 0.5 *p* = 0.03	0 ± 0.4 *p* = 0.68	*p* = 0.16
Alipour 2014 [36]	Iran	Randomized, double-blind, placebo-controlled clinical trial	RA	Probiotics	*Lactobacillus casei* 01, 10^8^ CFU per capsule, taken daily, 8 weeks	22	24	**CRP**	**Decreased**	**0.10 ± 0.79** ***p* = 0.304**	**0.95 ± 2.06** ***p* = 0.001**	***p* = 0.009**
								IL-1β	Decreased	2.13 ± 0.02	6.22 ± 0.10	*p* = 0.198
								IL-6	Decreased	24.65 ± 0.01	0.73 ± 0.01	*p* = 0.326
								**IL-12**	**Decreased**	**39.28 ± 1.41**	**35.61 ± 4.70**	***p* = 0.00**
								**IL-10**	**Increased**	**2.43 ± 8.02**	**1.51 ± 3.23**	***p* = 0.007**
								**TNF-α**	**Decreased**	**0.05 ± 0.00**	**1.76 ± 1.12**	***p* = 0.002**
Zamani 2017 [37]	Iran	Randomized, double-blind, placebo-controlled clinical trial	RA	Synbiotics	Synbiotic capsule containing *Lactobacillus acidophilus*, *Lactobacillus casei, and Bifidobacterium bifidum* (2 × 10^9^ CFU each) with 800 mg inulin, 8 weeks	27	27	TAC	Increased	11.1 ± 198.6	41.4 ± 71.2	*p* = 0.45
								**GSH**	**Increased**	**58.5 ± 154.4**	**36.6 ± 63.5**	***p* = 0.005**
								**CRP**	**Decreased**	**2833.4 ± 5639.7** ***p* = 0.01**	**1427.8 ± 3267.2** ***p* = 0.03**	***p* = 0.001**
								**NO**	**Increased**	**2.6 ± 4.5** ***p* = 0.006**	**0.8 ± 4.4** ***p* = 0.36**	***p* = 0.008**
								MDA	Decreased	0.1 ± 0.4 *p* = 0.05	0.1 ± 0.4 *p* = 0.73	*p* = 0.07
Esmaeili 2020 [38]	Iran	Randomized, double-blind, placebo-controlled clinical trial	RA	Synbiotics	Daily synbiotic capsule (Familact^®^) containing prebiotic fructooligosaccharides and probiotics (*Lactobacillus acidophilus*, *L. bulgaricus*, *L. casei*, *L. rhamnosus, Bifidobacterium breve*, *B. longum*, and *Streptococcus thermophiles* at 10^9^ CFU/mL per strain), 3 months	88	98	**CRP**	**Decreased**	**0.23 ± 0.23**	**2.11 ± 1.04**	***p* < 0.05**
								ESR	Decreased	0.19 ± 0.20	2.83 ± 0.60	*p* > 0.05
Asghari 2023 [39]	Iran	Randomized, double-blinded, placebo-controlled clinical trial	MS	Probiotics	*Saccharomyces boulardii* (250 mg, 10^10^ CFU) in BioDigest^®^ capsules, taken daily, 4 months	20	20	TAC	Increased	0.06 ± 1.22	0.51 ± 1.33	*p* = 0.163
								**CRP**	**Decreased**	**0.38 ± 1.42**	**2.26 ± 2.60**	***p* = 0.001**
								MDA	Decreased	0.43 ± 1.12	0.54 ± 1.15	*p* = 0.774
Salami 2019 [40]	Iran	Randomized, double-blind, placebo-controlled clinical trial	MS	Probiotics	Multi-strain probiotic supplement containing *Lactobacillus acidophilus*, *Bifidobacterium bifidum*, *Lactobacillus casei*, and *Lactobacillus fermentum*; dosage: 2 × 10^9^ CFU each, administered daily, 4 months	24	24	**CRP**	**Decreased**	**1.07 ± 0.5**	**0.61 ± 0.58**	***p* = 0.03**
								**NO**	**Increased**	**1.64 ± 1.27**	**2.87 ± 1.16**	***p* = 0.012**
								**IL-10**	**Increased**	**0.3 ± 0.22**	**0.46 ± 0.16**	***p* < 0.001**
								**IL-6**	**Decreased**	**0.07 ± 0.08**	**0.2 ± 0.1**	***p* = 0.01**
								TNF-α	decreased	0.21 ± 0.3	0.19 ± 0.11	*p* = 0.21
								SOD	Increased	3.37 ± 8.46	7.92 ± 8.55	*p* = 0.7
								GSH	Increased	6.36 ± 13.11	41.65 ± 16.84	*p* = 0.1
								TAC	Increased	15.48 ± 11.96	2.05 ± 9.81	*p* = 0.26
								**MDA**	**Decreased**	**0.15 ± 0.079**	**0.31 ± 0.075**	***p* < 0.001**
Rahimlou 2022 [41]	Iran	Randomized, double-blind, placebo-controlled clinical trial	MS	Probiotics	Multi-strain probiotic containing *Bacillus subtilis*, *Bifidobacterium bifidum*, *Bifidobacterium breve*, *Bifidobacterium infantis, Bifidobacterium longum*, *Lactobacillus acidophilus*, *Lactobacillus delbrueckii, Lactobacillus casei*, *Lactobacillus plantarum*, *Lactobacillus rhamnosus*, *Lactobacillus helveticus*, *Lactobacillus salivarius, Lactococcus lactis*, and *Streptococcus*, 12 weeks of thermophilus	33	32	**CRP**	**Decreased**	**0.05 ± 1.74** ***p* = 0.71**	**0.93 ± 1.62** ***p* = 0.04**	***p* = 0.03**
								**TNF-α**	**Decreased**	**0.48 ± 2.53** ***p* = 0.367**	**2.09 ± 1.88** ***p* = 0.021**	***p* = 0.015**
								**IFN-γ**	**Decreased**	**1.93 ± 5.99** ***p* = 0.12**	**13.18 ± 7.33** ***p* < 0.001**	***p* < 0.001**
								IL-17	Increased	1.32 ± 1.97 *p* = 0.18	0.02 ± 1.19 *p* = 0.32	*p* = 0.19
								**IL-6**	**Decreased**	**0.65 ± 2.21** ***p* < 0.001**	**6.70 ± 3.12** ***p* < 0.001**	***p* < 0.001**
Widhani 2022 [42]	Indonesia	Randomized, double-blind, placebo-controlled trial	SLE	Synbiotics	Synbiotic capsule containing *Lactobacillus helveticus R0052* (60%), *Bifidobacterium infantis R0033* (20%), *Bifidobacterium bifidum R0071* (20%) totaling 3 × 10^9^ CFU, and 80 mg fructo-oligosaccharides, 60 days	23	23	**CRP**	**No change**	**2.0 ± 5.7** ***p* = 0.005**	**0.3 ± 3.8** ***p* = 0.23**	***p* = 0.002**
								IL-6	Decreased	0.57 ± 13.85 *p* = 0.78	2.17 ± 4.19 *p* = 0.02	*p* = 0.27
								IL-17	No change	0 ± 1.25 *p* = 0.5	0.43 ± 0.69 *p* = 0.9	*p* = 0.6
Wang 2022 [43]	Taiwan	Randomized, double-blind, placebo-controlled clinical trial	T1DM	Probiotics	Daily probiotic capsule containing *Lactobacillus salivarius* subsp. *salicinius AP-32*, *Lactobacillus johnsonii MH-68*, *Bifidobacterium animalis* subsp. lactis CP-9 (5 × 10^9^ CFU per capsule), 6 months	27	29	**IL-8**	**Decreased**	**10 ± 317.0**	**47.8 ± 324.7** ***p* < 0.001**	***p* < 0.01**
								**TNF-α**	**Decreased**	**4.7 ± 80.6**	**13.3 ± 76.3** ***p* < 0.001**	***p* < 0.05**
								**IL-17**	**Decreased**	**0.4 ± 34.5**	**7.1 ± 40.3** ***p* < 0.01**	***p* < 0.05**
								**MIP-1b**	**Decreased**	**1.8 ± 121.9**	**14.3 ± 107.7** ***p* < 0.01**	***p* < 0.05**
								RANTES	Decreased	13.8 ± 91.7	46.2 ± 103.7 *p* < 0.01	Not significant between placebo and intervention group
Maleki 2023 [44]	Iran	Randomized, double-blind, placebo-controlled trial	Axial Spondyloarthritis	Synbiotics	Synbiotic capsule containing *Lacticaseibacillus casei*, *Lactobacillus acidophilus*, *Lactobacillus rhamnosus*, *Lactobacillus bulgaricus*, *Bifidobacterium longum*, *Bifidobacterium breve*, *and Streptococcus thermophilus* (10^9^ CFUs) along with fructooligosaccharides, 12 weeks	19	19	IL-17	Decreased	5.89 ± 19.90 *p* = 0.188	13.84 ± 18.54 *p* = 0.002	*p* = 0.057
								IL-23	Decreased	5.72 ± 20.73 *p* = 0.0100	19.61 ± 21.42 *p* < 0.001	*p* = 0.06
								CRP	Decreased	0.12 ± 1.33 *p* = 0.503	0.13 ± 1.41 *p* = 0.472	*p* = 0.903
Ou 2021 [45]	China	Randomized, placebo-controlled trial	UC	Probiotics	Probiotics supplement (*Bifidobacterium* triple viable capsule, 420 mg taken three times daily) combined with WeChat-based health management, 12 weeks	69	73	**IL-6**	**Decreased**	**8.49 ± 4.57**	**13.76 ± 4.31**	***p* < 0.05**
								**IL-8**	**Decreased**	**9.57 ± 13.28** ***p* < 0.05**	**14.44 ± 12.54** ***p* < 0.05**	***p* < 0.05**
								**TNF-α**	**Decreased**	**9.6 ± 5.23** ***p* < 0.05**	**11.3 ± 4.94** ***p* < 0.05**	***p* < 0.05**
Hegazy 2010 [46]	Egypt	Randomized, controlled clinical trial	UC	Probiotics	Probiotic preparation containing *Lactobacillus delbruekii* and *Lactobacillus fermentum*, 10 billion CFU per sachet, dissolved in water and taken with sulfasalazine (2400 mg daily), 8 weeks	15	15	IL-6	Decreased	*p* < 0.05	*p* < 0.05	Not reported
Groeger 2013 [47]	Ireland	Randomized, double-blind, placebo-controlled clinical trial	UC	Probiotics	Daily dose of *Bifidobacterium infantis* 35624, 1 × 10^10^ CFU, administered as a sachet in powder form, 8 weeks	22	35	**CRP**	**Decreased**	**27.29 ± 24.17**	**1.99 ± 4.24**	***p* = 0.0327**
								IL-6	Decreased	2.29 ± 1.91	0.19 ± 0.80	*p* = 0.057
								TNF-α	Decreased	0.21 ± 1.27	2.26 ± 0.65	Not significant between placebo and intervention group
Oliva 2012 [48]	Italy	Randomized, double-blind, placebo-controlled clinical trial	UC	Probiotics	Rectal enema containing *Lactobacillus reuteri* ATCC 55730 at 10^10^ CFU, administered in addition to oral mesalazine, 8 weeks	20	20	IL-1β	Decreased	No numbers reported	No numbers reported *p* < 0.01	Not significant between placebo and intervention group
								IL-8	Decreased	No numbers reported	No numbers reported *p* < 0.01	Not significant between placebo and intervention group
								TNF-α	Decreased	No numbers reported	No numbers reported *p* < 0.01	Not significant between placebo and intervention group
								IL-10	Increased	No numbers reported	No numbers reported *p* < 0.01	Not significant between placebo and intervention group
Cui 2004 [49]	China	Randomized, placebo-controlled clinical trial	UC	Probiotics	Bifid triple viable capsule (BIFICO) containing *Bifidobacterium*, *Enterococcus*, and *Lactobacillus* strains at a dose of 1.26 g/day, 8 weeks	15	15	IL-10	Increased	0.09 ± 0.26	0.35 ± 0.38 *p* < 0.01	Not reported
								TNF-α	Decreased	0.54 ± 0.19	0.44 ± 0.13 *p* < 0.01	Not reported
								IL-1β	Increased	0.82 ± 0.14	0.12 ± 0.14	Not reported
Bamola 2022 [50]	India	Randomized, double-blind, placebo-controlled clinical trial	UC	Probiotics	*Bacillus clausii* UBBC-07, administered at a dose of 2 billion CFU per capsule, taken twice daily with standard medical treatment (SMT), 4 weeks	54	54	IL-10	Increased	0.90 ± 5.98	9.90 ± 5.00 *p* < 0.05	Not reported
								IL-6	Decreased	4.8 ± 7.48	13.1 ± 10.28 *p* < 0.05	Not reported
								IL-17	Decreased	3.1 ± 7.72	14.5 ± 7.74 *p* < 0.05	Not reported
								IL-23	Decreased	9.78 ± 100.34	92.9 ± 71.87	Not reported
								IL-1β	Decreased	34.2 ± 87.87	138 ± 59.56 *p* < 0.05	Not reported
								TNF-α	Decreased	6.5 ± 10.83	7.3 ± 8.36	Not reported
Altun 2019 [51]	Turkey	Randomized, placebo-controlled clinical trial	UC	Synbiotics	Synbiotic chewable tablets containing *Enterococcus faecium*, *Lactobacillus plantarum*, *Streptococcus thermophilus*, *Bifidobacterium lactis*, *Lactobacillus acidophilus*, *Bifidobacterium longum* (3 × 10^9^ CFU), and 225 mg of fructooligosaccharide (prebiotic fiber) per tablet, taken twice daily, 8 weeks	18	18	CRP	Decreased	0.2 ± 1.65 *p* = 0.170	0.3 ± 1.28 *p* = 0.003	*p* = 0.051
								ESR	Decreased	1.3 ± 15.06 *p* = 0.740	15.7 ± 35.47 *p* = 0.003	*p* = 0.137
Wang 2023 [52]	China	Randomized, controlled clinical trial	UC	FMT	IMT via transscopic intestinal implantation weekly combined with oral mesalamine (four enteric tablets taken three times daily), 30 days	47	47	**TNF-α**	**decreased**	**0.46 ± 1.08**	**1.04 ± 1.02**	***p* < 0.05**
								**IL-1β**	**decreased**	**5.82 ± 3.68**	**9.54 ± 3.77**	***p* < 0.05**
								**IL-17**	**decreased**	**123.10 ± 85.55**	**257.26 ± 90.62**	***p* < 0.05**
								**IL-23**	**decreased**	**419.47 ± 123.53**	**524.85 ± 111.00**	***p* < 0.05**
Bourreille 2013 [53]	France	Randomized, double-blind, placebo-controlled clinical trial	CD	Probiotics	*Saccharomyces boulardii* administered at 1 g per day, 52 weeks	84	81	CRP	No change	0.7 (no SD reported)	2.5 (no SD reported)	Not significant between placebo and intervention group
								ESR	No change	0.8 ± 19.0 (no SD reported)	1.8 (no SD reported)	Not significant between placebo and intervention group
Shen 2024 [54]	China	Randomized, controlled clinical trial	CD	Probiotics	Mesalamine (1 g three times daily) combined with *Bifidobacterium*, *Lactobacillus*, and *Enterococcus* capsules (0.42 g three times daily), 4 weeks	48	48	**TNF-α**	**Decreased**	**11.1 (no SD reported)** ***p* < 0.05**	**17.9 (no SD reported)** ***p* < 0.05**	***p* < 0.05**
								**IL-6**	**Decreased**	**5.7 (no SD reported)** ***p* < 0.05**	**10.6 (no SD reported)** ***p* < 0.05**	***p* < 0.05**
								**IL-10**	**Increased**	**3.3 (no SD reported)** ***p* < 0.05**	**6.4 (no SD reported)** ***p* < 0.05**	***p* < 0.05**
								**CRP**	**Decreased**	**4.3 (no SD reported)** ***p* < 0.05**	**8.2 (no SD reported)** ***p* < 0.05**	***p* < 0.05**
Bamola 2022 [50]	India	Randomized, double-blind, placebo-controlled clinical trial	CD	Probiotics	*Bacillus clausii* UBBC-07, administered at a dose of 2 billion CFU per capsule, taken twice daily with standard medical treatment (SMT), 4 weeks	54	54	IL-10	Increased	3.40 ± 5.49	10.00 ± 4.88 *p* < 0.05	None reported
								IL-6	Decreased	3.2 ± 7.37	6.0 ± 8.65	None reported
								IL-17	Decreased	2.2 ± 14.14	16.8 ± 8.82 *p* < 0.05	None reported
								IL-23	Decreased	35.9 ± 87.17	68 ± 63.39	None reported
								IL-1β	Decreased	160.5 ± 75.77	107.2 ± 48.59 *p* < 0.05	None reported
								TNF-α	Decreased	9.3 ± 16.59	13.1 ± 10.28	None reported
Fan 2019 [55]	China	Randomized, placebo-controlled clinical trial	Mixed IBD	Probiotics	Patients in the observation group received two probiotic tablets (Bifico, containing *Bifidobacterium*, *Enterococcus*, and *Lactobacillus* species) three times daily, combined with Pentasa (mesalazine) tablets at 1–2 tablets, three times a day, 8 weeks	21	19	CRP	Decreased	No numbers reported	No numbers reported	*p* = 0.05
								IL-6	Decreased	No numbers reported	No numbers reported	*p* = 0.05
Shadnoush 2013 [56]	Iran	Randomized, double-blind, placebo-controlled clinical trial	Mixed IBD	Probiotics	250 g of probiotic yogurt containing *Bifidobacterium* and *Lactobacillus strains* (1.5% fat) daily, 8 weeks	86	90	**CRP**	**Decreased**	**0.2 ± 4.96** ***p* < 0.05**	**2.2 ± 4.98** ***p* < 0.01**	***p* < 0.001**
								**IL-1β**	**Decreased**	**3 ± 4.6** ***p* < 0.05**	**24 ± 5.02** ***p* < 0.01**	***p* < 0.001**
								**TNF-α**	**Decreased**	**4 ± 10.51** ***p* > 0.05**	**90 ± 10.33** ***p* < 0.001**	***p* < 0.001**
								**IL-10**	**Increased**	**3.00 ± 1.75** ***p* < 0.05**	**27.00 ± 3.62** ***p* < 0.001**	***p* < 0.001**
								**IL-6**	**Increased**	**2 ± 3.74** ***p* < 0.05**	**17 ± 3.47** ***p* < 0.01**	***p* < 0.001**
Talebi 2020 [57]	Iran	Randomized, double-blind, placebo-controlled trial	Hypothyroidism	Synbiotics	Synbiotic capsule containing *Lactobacillus* and *Bifidobacterium* species (7 × 10^9^ CFU of *L. casei*, *L. acidophilus*, *L. rhamnosus*, *L. bulgaricus*, *B. breve*, *B. longum*, and *Streptococcus thermophilus*), plus fructooligosaccharide as a prebiotic, taken daily (500 mg), 8 weeks	29	27	CRP	Increased	0.34 ± 0.29 *p* = 0.25	0.58 ± 0.19 *p* = 0.006	*p* = 0.699
Shukla 2016 [58]	India	Randomized, double-blind, placebo-controlled clinical trial	ERA/JIA	Probiotics	Probiotic VSL#3 *containing Streptococcus thermophilus*, *Bifidobacterium breve*, *B. longum*, *B. infantis*, *Lactobacillus acidophilus*, *L. plantarum*, *L. paracasei*, *L. delbrueckii*, administered twice daily, totaling 112.5 billion CFUs per day, 12 weeks	23	23	ESR	Decreased	50 ± 23.3 *p* < 0.01	38 ± 32.18	*p* = 0.35
								CRP	Decreased	1.7 ± 1.43 *p* < 0.01	7.3 ± 2.94 *p* < 0.05	*p* = 0.36
								IFN-γ	No change	0 ± 0.2	0 ± 1.1	*p* = 0.5
								IL-4	No change	0 ± 0.64	0 ± 1.71	*p* = 0.3
								IL-6	Decreased	19.5 ± 22.8	41.6 ± 22.2 *p* < 0.01	*p* = 0.13
								IL-17	Decreased	19.7 ± 22.36	2 ± 29.89	*p* = 0.26
								**IL-10**	**Decreased**	**1.0 ± 1.25**	**0.75 ± 1.30**	***p* = 0.013**
								TNF-α	Decreased	0.2 ± 0.36	0.49 ± 2.29	*p* = 0.5
Groeger 2013 [47]	Ireland	Randomized, double-blind, placebo-controlled clinical trial	Psoriasis	Probiotics	Daily dose of *Bifidobacterium infantis* 35,624, 1 × 10^10^ CFU, administered as a sachet in powder form, 8 weeks	26	35	**CRP**	**Decreased**	**0.87 ± 1.12**	**1.58 ± 1.38**	***p* = 0.0425**
								IL-6	Decreased	0.31 ± 0.50	0.14 ± 0.54	Not significant between placebo and intervention group
								**TNF-α**	**Decreased**	**0.34 ± 0.76**	**0.53 ± 1.09**	***p* = 0.0405**
Moludi 2022 [59]	Iran	Randomized, double-blind, placebo-controlled clinical trial	Psoriasis	Probiotics	Multi-strain probiotic containing *Lactobacillus acidophilus*, *Bifidobacterium bifidum*, *Bifidobacterium lactis*, and *Bifidobacterium* longum at a minimum concentration of 1.6 × 10^9^ CFU/g. Administered twice daily for a total of 8 weeks, 8 weeks	23	23	**CRP**	**Decreased**	**0.70 ± 1.61** ***p* = 0.111**	**1.67 ± 1.22** ***p* = 0.001**	***p* = 0.013**
								**IL-1β**	**Decreased**	**0.2 ± 1.52** ***p* = 0.829**	**1.64 ± 2.04** ***p* = 0.003**	***p* = 0.043**
								**LPS**	**Decreased**	**2.74 ± 13.1** ***p* = 0.498**	**7.21 ± 8.51** ***p* = 0.019**	***p* = 0.010**

### 3.3. CRP Meta-Analysis

#### 3.3.1. Overall Effect

**Figure 3:**
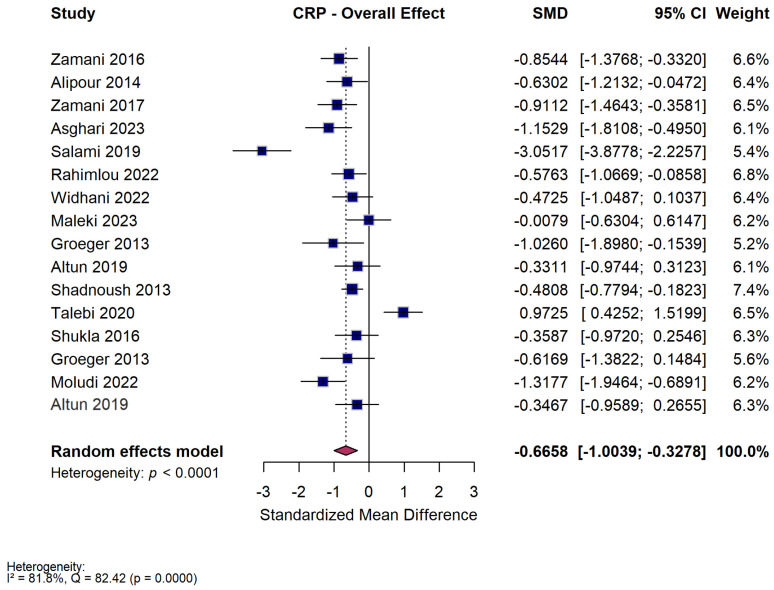
Forest plot of the SMD in CRP levels comparing probiotic interventions to control across included studies. Boxes represent each study’s effect size, with horizontal lines indicating 95% confidence intervals. The diamond depicts the pooled random effects estimate and its 95% confidence interval [35,36,37,39,40,41,42,44,47,51,56,57,58,59].

Sixteen studies reported data on CRP sufficient to be included in the meta-analysis. Across the included trials, probiotic and synbiotic supplementation was associated with a statistically significant reduction in circulating CRP compared with control groups, as seen by the SMD (SMD −0.67 [95% CI −1.00 to −0.33, *p* < 0.001]) (Figure 3). Although heterogeneity was substantial (I^2^ = 80.8%), the direction of effect consistently favored the intervention arms. This indicates that microbial supplementation exerts a measurable anti-inflammatory effect reflected by lower CRP concentrations. Six studies that reported on CRP had medium risk bias (Zamani 2016 [35]—D1, D2, D4; Alipour 2014 [36]—D1; Zamani 2017 [37]—D4; Groeger 2013 [47]—D1; Shadnoush 2013 [56]—D3). There were no high risk studies to exclude in order to perform a sensitivity analysis.

#### 3.3.2. Subgroup Analysis—Intervention Type

When trials were grouped by intervention type, differences in magnitude were observed. Multi-strain probiotic formulations showed the largest reduction in CRP (SMD −1.04 [95% CI −1.63 to −0.45, *p* < 0.001, I^2^ = 87%]), followed by single-strain probiotics (SMD −0.84 [95% CI −1.18 to −0.49, *p* < 0.001, I^2^ = 0%]) (Supplementary Figure S2A). Synbiotic interventions, in contrast, did not produce a significant change (SMD −0.18 [95% CI −0.72 to 0.36, *p* = 0.52, I^2^ = 80%]). The test for subgroup differences was not significant (Q = 5.36, *p* = 0.07). Taken together, these findings suggest that probiotics alone, particularly multi-strain products, are more effective than synbiotics in reducing CRP in ADs.

#### 3.3.3. Subgroup Analysis—Duration of Intervention

Duration appeared to influence the magnitude of effect. Shorter interventions (≤8 weeks) resulted in a moderate but significant decrease in CRP (SMD −0.53 [95% CI −0.87 to −0.19, *p* = 0.002, I^2^ = 75%]), while longer interventions (>8 weeks) produced a greater numerical reduction (SMD −1.00 [95% CI −1.87 to −0.12, *p* = 0.025, I^2^ = 90%]) (Supplementary Figure S2B). The difference between duration subgroups was not statistically significant (Q = 0.95, *p* = 0.33). Nonetheless, the trend toward a larger reduction with extended treatment suggests that prolonged probiotic use may strengthen anti-inflammatory responses over time.

#### 3.3.4. Subgroup Analysis—Dose

Results were consistent across dose categories. Low-dose regimens significantly lowered CRP (SMD −0.66 [95% CI −1.12 to −0.21, *p* = 0.004, I^2^ = 87%]), and high-dose regimens achieved a similar effect (SMD −0.76 [95% CI −1.14 to −0.37, *p* < 0.001, I^2^ = 14%]) (Supplementary Figure S2C). The test for subgroup differences was not significant (Q = 0.10, *p* = 0.76). These data indicate that the anti-inflammatory benefit of probiotics is not dose-dependent within the ranges studied, and that moderate doses may be adequate to achieve clinically meaningful improvements.

#### 3.3.5. Subgroup Analysis—Disease

Exploratory subgroup analysis by disease showed variation in the degree of CRP reduction across autoimmune conditions. Significant decreases were seen in MS (SMD = −1.56 [95% CI −2.89 to −0.23, *p* = 0.022, I^2^ = 92%]), psoriasis (SMD = −1.00 [95% CI −1.69 to −0.32, *p* = 0.004, I^2^ = 48%]), RA (SMD = −0.81 [95% CI −1.12 to −0.49, *p* < 0.001, I^2^ = 0%]), and UC (SMD = −0.48 [95% CI −0.88 to −0.09, *p* = 0.017, I^2^ = 0%]) (Supplementary Figure S2D). Smaller, non-significant effects were observed in ankylosing spondyloarthritis, juvenile idiopathic arthritis, and SLE. One study in hypothyroidism reported an increase in CRP (SMD = 0.97 [95% CI 0.43 to 1.52, *p* < 0.001]). The test for subgroup differences was statistically significant (Q = 38.03, *p* < 0.001), indicating variability among diseases. Because most subgroups were represented by only one or two studies, these results should be considered exploratory and interpreted with caution rather than as evidence of true between-disease differences.

### 3.4. IL-6 Meta-Analysis

#### 3.4.1. Overall Effect

**Figure 4:**
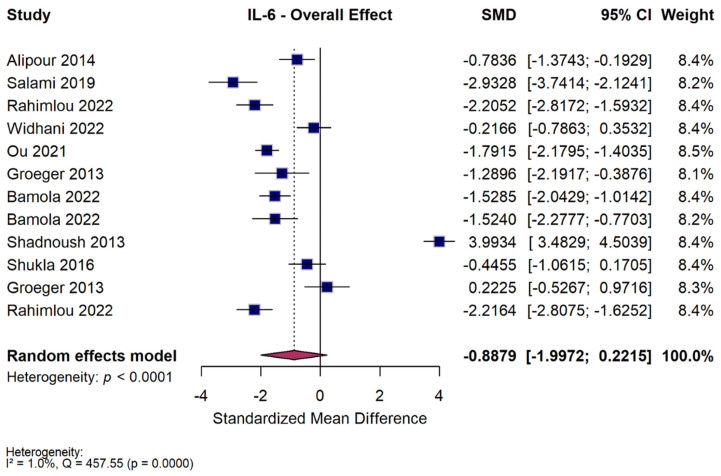
Forest plot of the SMD in IL-6 levels comparing probiotic interventions to control across included studies. Boxes represent each study’s effect size, with horizontal lines indicating 95% confidence intervals. The diamond depicts the pooled random effects estimate and its 95% confidence interval [36,40,41,42,45,47,50,56,58].

Twelve studies assessed IL-6 as an outcome and were included in the meta-analysis. Across the included trials, probiotic and synbiotic supplementation did not significantly reduce circulating IL-6 concentrations compared with controls (SMD = −0.89 [95% CI −1.99 to 0.22, *p* = 0.12, I^2^ = 98%]) (Figure 4). The wide confidence interval and substantial heterogeneity indicated marked variability among studies, suggesting that the overall effect of microbial supplementation on IL-6 was inconsistent. While several individual trials reported decreases in IL-6, the pooled estimate was not statistically significant. These findings show no consistent or significant overall effect of probiotics on IL-6 levels. Three studies had medium risk of bias (Alipour 2014 [36]—D1; Groeger 2013 [47]—D1; Shadnoush 2013 [56]—D3) and one had high risk of bias (Bamola 2022 [50]—D3). In sensitivity analyses, excluding the study assessed as high risk of bias, the pooled effect estimate for IL-6 was attenuated and not statistically significant (SMD = −0.76, 95% CI −2.08 to 0.56; *p* = 0.259), with minimal heterogeneity among the remaining studies (I^2^ = 1%)

#### 3.4.2. Subgroup Analysis—Intervention Type

When studies were stratified by intervention type, single-strain probiotics significantly reduced IL-6 (SMD = −0.99 [95% CI −1.62 to −0.36, *p* = 0.002, I^2^ = 76%]), whereas multi-strain probiotics showed a nonsignificant change (SMD = −0.93 [95% CI −3.05 to 1.20, *p* = 0.39, I^2^ = 99%]) (Supplementary Figure S3A). The single synbiotic trial also showed no significant effect (SMD = −0.22 [95% CI −0.79 to 0.35, *p* = 0.46]). The test for subgroup differences was not statistically significant (Q = 3.28, *p* = 0.19). Therefore, IL-6 reduction did not differ significantly between probiotic and synbiotic interventions.

#### 3.4.3. Subgroup Analysis—Duration of Intervention

Duration appeared to influence outcomes. Short-term interventions (≤8 weeks) showed no measurable change (SMD = 0.03 [95% CI −1.82 to 1.88, *p* = 0.97, I^2^ = 98%]), whereas longer interventions (>8 weeks) produced a significant reduction in IL-6 (SMD = −1.81 [95% CI −2.43 to −1.18, *p* < 0.001, I^2^ = 84%]) (Supplementary Figure S3B). The difference between duration subgroups was not statistically significant (Q = 3.40, *p* = 0.07). This indicates that intervention length did not significantly modify the overall effect on IL-6.

#### 3.4.4. Subgroup Analysis—Dose

Subgroup analysis by dose showed no significant effect at either treatment level. Low-dose regimens resulted in a nonsignificant change (SMD = −0.87 [95% CI −2.42 to 0.68, *p* = 0.27, I^2^ = 98%]), and high-dose regimens showed a similar pattern (SMD = −0.47 [95% CI −1.24 to 0.30, *p* = 0.23, I^2^ = 69%]) (Supplementary Figure S3C). The comparison between subgroups was not statistically significant (Q = 0.20, *p* = 0.65). Accordingly, probiotic dose did not significantly influence IL-6 outcomes.

#### 3.4.5. Subgroup Analysis—Disease

Exploratory subgroup analysis revealed marked variability in IL-6 response across different autoimmune conditions. Significant reductions in IL-6 were observed in MS (SMD = −2.38 [95% CI −2.79 to −1.96, *p* < 0.001, I^2^ = 17%]), UC (SMD = −1.65 [95% CI −1.95 to −1.36, *p* < 0.001, I^2^ = 0%]), Crohn’s disease (SMD = −1.52 [95% CI −2.28 to −0.77, *p* < 0.001]), and RA (SMD = −0.78 [95% CI −1.37 to −0.19, *p* = 0.009]) (Supplementary Figure S3D). No significant effects were seen in psoriasis, SLE, juvenile idiopathic arthritis, or other individual subgroups. One study in inflammatory bowel disease reported a marked increase in IL-6 (SMD = 3.99 [95% CI 3.48 to 4.50, *p* < 0.001]), which may reflect disease-specific variability or measurement context rather than a true pro-inflammatory effect. The test for subgroup differences was statistically significant (Q = 442.02, *p* < 0.001), indicating high heterogeneity among disease categories. Similarly to the disease analysis in CRP, most subgroups were represented by single studies, so these results should be viewed as exploratory and interpreted with caution.

### 3.5. IL-10 Meta-Analysis

#### 3.5.1. Overall Effect

**Figure 5:**
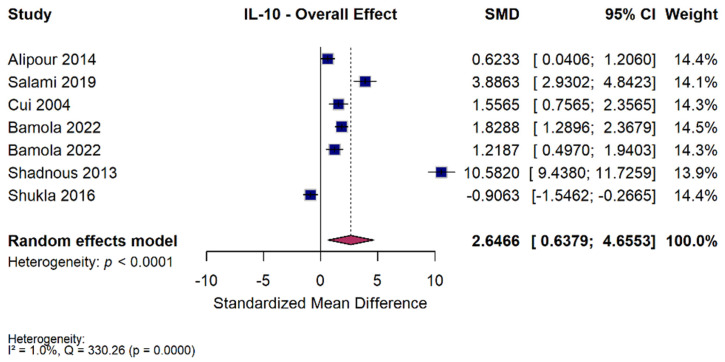
Forest plot of the SMD in IL-10 levels comparing probiotic interventions to control across included studies. Boxes represent each study’s effect size, with horizontal lines indicating 95% confidence intervals. The diamond depicts the pooled random effects estimate and its 95% confidence interval [36,40,49,50,56,58].

Ten studies were included in the IL-10 meta-analysis. Across the included studies, probiotic and synbiotic supplementation significantly increased circulating IL-10 levels compared with controls (SMD = 2.65 [95% CI 0.64 to 4.66, *p* = 0.010, I^2^ = 98%]) (Figure 5). Although heterogeneity was substantial, the direction of effect was consistent across most studies, suggesting a general enhancement of anti-inflammatory cytokine production following microbial intervention. These results indicate that probiotics can meaningfully upregulate IL-10, a key anti-inflammatory mediator. Two studies had medium risk of bias (Alipour 2014 [36]—D1; Shadnoush 2013 [56]—D3), and one had high risk of bias (Bamola 2022 [50]—D3). In sensitivity analyses, excluding the study assessed as high risk of bias, the pooled effect estimate for IL-10 was no longer statistically significant (SMD = 3.12, 95% CI −0.02 to 6.26; *p* = 0.052), with minimal heterogeneity (I^2^ = 1%)

#### 3.5.2. Subgroup Analysis—Intervention Type

When studies were stratified by intervention type, single-strain probiotics produced a significant increase in IL-10 (SMD = 1.23 [95% CI 0.49 to 1.97, *p* = 0.001, I^2^ = 77%]), while multi-strain probiotics showed a larger but non-significant pooled effect (SMD = 3.76 [95% CI −0.58 to 8.11, *p* = 0.090, I^2^ = 99%]) (Supplementary Figure S4A). The test for subgroup differences was not statistically significant (Q = 1.27, *p* = 0.26). Thus, while both single- and multi-strain formulations tended to raise IL-10, the difference between them was not significant.

#### 3.5.3. Subgroup Analysis—Duration of Intervention

Duration did not appear to significantly influence outcomes. Shorter interventions (≤8 weeks) were associated with a significant rise in IL-10 (SMD = 3.12 [95% CI 0.66 to 5.58, *p* = 0.013, I^2^ = 98%]), whereas longer interventions (>8 weeks) produced a smaller and statistically non-significant increase (SMD = 1.48 [95% CI −3.22 to 6.17, *p* = 0.54, I^2^ = 99%]) (Supplementary Figure S4B). The comparison between duration subgroups was not significant (Q = 0.37, *p* = 0.54). These findings suggest that treatment length did not significantly modify IL-10 outcomes.

#### 3.5.4. Subgroup Analysis—Dose

Subgroup analysis by dose revealed a differential effect. Low-dose interventions significantly increased IL-10 (SMD = 3.59 [95% CI 0.99 to 6.18, *p* = 0.007, I^2^ = 98%]), while high-dose interventions were associated with a reduction (SMD = −0.91 [95% CI −1.55 to −0.27, *p* = 0.005]) (Supplementary Figure S4C). The difference between dose subgroups was statistically significant (Q = 10.86, *p* < 0.001), indicating that higher doses may not confer additional benefit and could potentially attenuate the IL-10 response. Overall, lower probiotic doses appeared more effective in enhancing IL-10 levels.

#### 3.5.5. Subgroup Analysis—Disease

Exploratory subgroup analysis by disease showed considerable variation in IL-10 response. Significant increases in IL-10 were observed in UC (SMD = 1.74 [95% CI 1.30 to 2.19, *p* < 0.001, I^2^ = 0%]), MS (SMD = 3.89 [95% CI 2.93 to 4.84, *p* < 0.001]), RA (SMD = 0.62 [95% CI 0.04 to 1.21, *p* = 0.036]), Crohn’s disease (SMD = 1.22 [95% CI 0.50 to 1.94, *p* < 0.001]), and inflammatory bowel disease (SMD = 10.58 [95% CI 9.44 to 11.73, *p* < 0.001]) (Supplementary Figure S4D). In contrast, one study in juvenile idiopathic arthritis reported a significant decrease (SMD = −0.91 [95% CI −1.55 to −0.27, *p* = 0.005]). The test for subgroup differences was statistically significant (Q = 329.96, *p* < 0.001), reflecting high variability between conditions. These results should be not seen as conclusive evidence of disease-specific differences.

### 3.6. TNF-α Meta-Analysis

#### 3.6.1. Overall Effect

**Figure 6:**
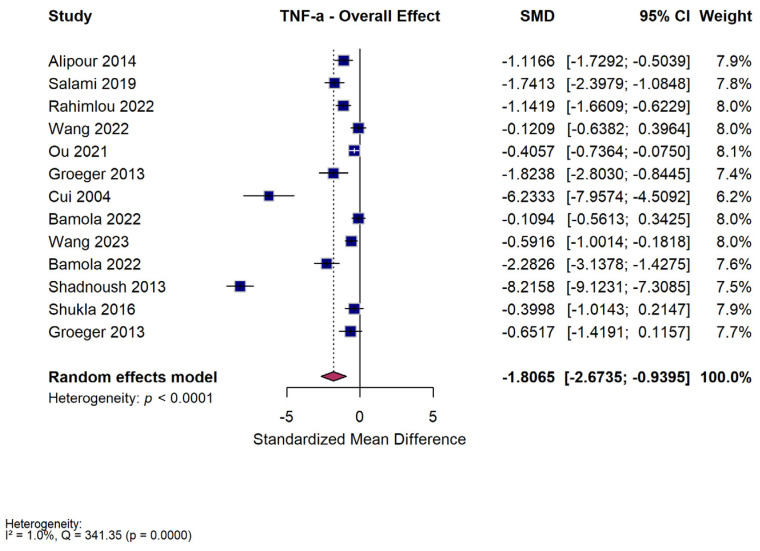
Forest plot of the SMD in TNF-*α* levels comparing probiotic interventions to control across included studies. Boxes represent each study’s effect size, with horizontal lines indicating 95% confidence intervals. The diamond depicts the pooled random effects estimate and its 95% confidence interval [36,40,41,43,45,47,49,50,52,56,58].

Thirteen studies were meta-analyzed on their reported TNF-*α* outcomes. Across the included studies, probiotic and microbiome-based interventions significantly reduced circulating TNF-α concentrations compared with controls (SMD = −1.81 [95% CI −2.67 to −0.94, *p* < 0.001, I^2^ = 96%]) (Figure 6). Despite considerable heterogeneity, the direction of effect consistently favored intervention groups. These findings indicate a robust overall anti-inflammatory effect of probiotics and related therapies on TNF-α. Three studies had medium risk of bias (Alipour 2014 [36]—D1; Groeger 2013 [47]—D1; Shadnoush 2013 [56]—D3), with one having high risk of bias (Bamola 2022 [50]—D3). In sensitivity analyses, excluding the study assessed as high risk of bias, the pooled effect estimate for TNF-α remained statistically significant (SMD = −1.94, 95% CI −2.94 to −0.93; *p* < 0.001), with minimal heterogeneity (I^2^ = 1%).

#### 3.6.2. Subgroup Analysis—Intervention Type

When stratified by intervention type, multi-strain probiotics showed the greatest reduction in TNF-α (SMD = −2.51 [95% CI −4.11 to −0.91, *p* = 0.002, I^2^ = 98%]), followed by single-strain probiotics (SMD = −1.14 [95% CI −1.93 to −0.35, *p* = 0.005, I^2^ = 85%]) and fecal microbiota transplantation (SMD = −0.59 [95% CI −1.00 to −0.18, *p* = 0.005]) (Supplementary Figure S5A). The test for subgroup differences was statistically significant (Q = 6.13, *p* = 0.047), suggesting variability in efficacy across intervention types. Overall, multi-strain probiotics appeared most effective in lowering TNF-α, although heterogeneity across studies remains high.

#### 3.6.3. Subgroup Analysis—Duration of Intervention

Treatment duration influenced the observed effects. Interventions lasting ≤8 weeks resulted in a larger pooled reduction in TNF-α (SMD = −2.67 [95% CI −4.43 to −0.91, *p* = 0.003, I^2^ = 98%]) than longer interventions lasting >8 weeks (SMD = −0.87 [95% CI −1.39 to −0.35, *p* < 0.001, I^2^ = 81%]) (Supplementary Figure S5B). The difference between subgroups was not statistically significant (Q = 3.69, *p* = 0.055). Thus, while shorter interventions showed numerically larger reductions, treatment duration did not significantly alter the overall TNF-α outcome.

#### 3.6.4. Subgroup Analysis—Dose

Subgroup analysis by dose showed that both low- and high-dose regimens were effective, but without significant difference between them. Low-dose interventions significantly reduced TNF-α (SMD = −1.85 [95% CI −3.10 to −0.60, *p* = 0.004, I^2^ = 98%]), while high-dose interventions yielded a smaller yet significant effect (SMD = −0.88 [95% CI −1.65 to −0.11, *p* = 0.025, I^2^ = 66%]) (Supplementary Figure S5C). The comparison between dose subgroups was not statistically significant (Q = 1.68, *p* = 0.19). These data suggest that higher probiotic doses did not produce additional TNF-α reduction within the ranges studied.

#### 3.6.5. Subgroup Analysis—Disease

Exploratory subgroup analysis demonstrated variability in TNF-α modulation across autoimmune conditions. Significant decreases were observed in inflammatory bowel disease (SMD = −8.22 [95% CI −9.12 to −7.31, *p* < 0.001]), Crohn’s disease (SMD = −2.28 [95% CI −3.14 to −1.43, *p* < 0.001]), MS (SMD = −1.41 [95% CI −1.99 to −0.82, *p* < 0.001, I^2^ = 49%]), RA (SMD = −1.12 [95% CI −1.73 to −0.50, *p* < 0.001]), and UC (SMD = −1.38 [95% CI −2.28 to −0.49, *p* = 0.002, I^2^ = 92%]) (Supplementary Figure S5D). Smaller or nonsignificant changes were reported in psoriasis, juvenile idiopathic arthritis, and T1DM. The test for subgroup differences was statistically significant (Q = 259.00, *p* < 0.001), reflecting marked heterogeneity across diseases. Given that several subgroups were based on single studies, these findings are more exploratory rather than confirmatory.

### 3.7. Additional Inflammatory Markers

Several studies also assessed additional markers beyond CRP, IL-6, IL-10, and TNF-α. In MS, Asghari et al. (2023) [39] reported a modest increase in total antioxidant capacity (TAC) following high-dose single-strain probiotic supplementation over 16 weeks (intervention +0.51 ± 1.33 vs. control −0.06 ± 1.22). In RA, Zamani et al. (2017) [37] found an increase in nitric oxide (NO) after 8 weeks of synbiotic therapy (intervention +0.8 ± 4.4 vs. control −2.6 ± 4.5), while T1DM and UC studies demonstrated consistent reductions in IL-8. Specifically, Wang et al. (2022) [60] observed a marked IL-8 decrease after 24 weeks of multi-strain probiotic treatment in T1DM (intervention -47.8 ± 265.8 vs. control −10 ± 201.6), and Ou et al. (2021) [45] reported similar findings in UC (intervention −14.4 ± 8.7 vs. control −9.6 ± 9.5). IL-17 changes were variable across studies: Wang et al. (2022) [60] showed a small reduction in T1DM (intervention −7.1 ± 44.6 vs. control −0.4 ± 33.8), while Maleki et al. (2023) [44] and Widhani et al. (2022) [42] reported modest increases in ankylosing spondyloarthritis following 12 weeks of synbiotic therapy. In contrast, Wang et al. (2023) [52] found a pronounced decrease in IL-17 in UC after 4 weeks of fecal microbiota transplantation (intervention −257.26 ± 75.26 vs. control −123.1 ± 75.73). Reductions in IL-23 were also observed, with Maleki et al. (2023) [44] noting decreased levels in ankylosing spondyloarthritis (intervention +51.8 ± 17.4 vs. control +32.2 ± 12.5), and Wang et al. (2023) [52] reporting substantial suppression in UC (intervention −524.85 ± 113.22 vs. control −419.47 ± 121.93). Meanwhile, Cui et al. (2004) [49] found only minor, nonsignificant reductions in IL-1β after 4 weeks of multi-strain probiotic therapy in UC (intervention +0.12 ± 0.13 vs. control +0.82 ± 0.14). Collectively, results of these studies have shown that microbial interventions modulate a range of inflammatory mediators and oxidative markers, particularly IL-8, IL-17, IL-23, NO, and TAC, though the magnitude and consistency of these effects vary across diseases and intervention types.

## 4. Discussion

**Figure 7:**
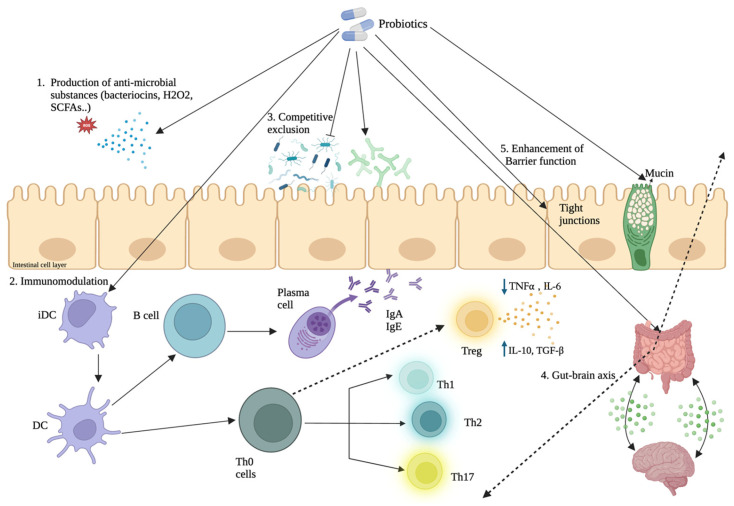
Mechanistic representation of probiotics functions in the gut, specifically at the intestinal epithelial barrier (created using BioRender, web-based version). This figure depicts the probiotic mechanism of action at the intestinal epithelial cell layer. Probiotics can interact with intestinal cells to exert immune responses. (1) Probiotics aid in the production of anti-microbial agents, such as bacteriocin and hydrogen peroxide. (2) Probiotics hypothetically modulate both innate and adaptive immune systems by interacting with dendritic cells (DCs), intestinal epithelial cells, and macrophages. DCs and macrophages detect metabolites produced by probiotics (e.g., short chain fatty acids (SCFAs)) via pattern recognition receptors, e.g., Toll-like receptor (TLR). Probiotic stimulation of these receptors enhances the production of anti-inflammatory cytokines (e.g., IL-10 and TGF-β) and, as a downstream effect, reduces pro-inflammatory cytokines such as IL-6 and TNF-α. Also, there is an upregulation of T regulatory cells with Th1 and Th2 balance and a reduction in Th17 cells. Dendritic cells induce maturation of B cells into plasma cells, which produce IgA. (3) Probiotics compete with pathogenic bacteria for the adhesion site at the epithelial layer to prevent pathogenic colonization. (4) In preclinical models, they alter the release of neurotransmitters from the gut, which allows for a cross-talk between the gut and the brain, highlighting its importance in neurological conditions. (5) Lastly, they enhance epithelial barrier integrity by strengthening tight junctions between epithelial cells, reducing intestinal permeability and preventing translocation of harmful pathogens or toxins. Solid arrows = direct/local probiotic effects; dashed arrows = indirect or downstream systemic pathways.

To the best of our knowledge, this meta-analysis comprehensively evaluates the effects of microbiome-modulating therapies on all of these inflammatory markers across this wide spectrum of multiple autoimmune diseases, along with oxidative stress markers, as a narrative review. Overall, this study demonstrated that these interventions were associated with significant reductions in key pro-inflammatory markers including CRP and TNF-α, alongside an increase in the anti-inflammatory cytokine IL-10. Among the interventions analyzed, multi-strain probiotic formulations showed the greatest effect on lowering TNF-α and CRP and increasing IL-10, while single-strain probiotics demonstrated notable improvements in IL-6. Synbiotics and FMT had more variable results but demonstrate notable, positive effects. By synthesizing evidence from 28 randomized controlled trials spanning 10 autoimmune conditions, this analysis provides the most comprehensive quantitative summary to date on the immunomodulatory potential of gut-targeted nutraceuticals in autoimmune disease. The potential mechanisms through which probiotics and related microbiome-modulating therapies exert immunomodulatory effects are summarized in Figure 7.

### 4.1. CRP

Our meta-analysis observed significant reductions in CRP, prompting us to examine the biological mechanisms behind the effect of these nutraceuticals. Interpretation of these pooled effects requires caution, as between-study heterogeneity was substantial to the extreme (I^2^ 80.8%), reflecting marked clinical and methodological diversity across diseases, interventions, and study designs. CRP is a downstream hepatic acute-phase reactant primarily regulated by IL-6 via the gp130-JAK-STAT3 axis, with additional input from TNF-α and IL-1β [17,18]. Elevated CRP in ADs reflects systemic inflammation, which is often linked to increased gut permeability and bacterial endotoxin movement that then drives the hepatic cytokine release. From the studies mentioned, nutraceutical supplementation appears to stop this harmful cascade by restoring intestinal barrier integrity and modulating innate immune signaling along the gut-liver axis. These mechanistic patterns align with our pooled analysis, which showed a significant overall reduction in CRP across 16 RCTs, suggesting that gut-modulating therapies could dampen systemic IL-6 and TNF-α driven inflammation. However, the heterogeneity brings hesitancy to draw firm conclusions regarding this.

Several strains or probiotics potentially lower CRP levels by interrupting the upstream inflammatory markers that trigger its hepatic synthesis. *Lactobacillus rhamnosus*, for example, is hypothesized to reduce CRP by strengthening the gut barrier and dampening upstream inflammation. Its p40 protein activates EGFR signaling, which increases tight-junction proteins and lowers gut permeability. This limits lipopolysaccharide (LPS) translocation, reducing TLR4–NF-κB (Nuclear factor kappa-light-chain-enhancer of activated B cells) activation and downstream IL-6 and TNF-α release, which are key drivers of hepatic CRP production via the STAT3 pathway [61]. This theory is consistent with the clinical trials from our meta-analysis using *L. rhamnosus* in psoriasis and mixed autoimmune cohorts, where this strain was a core component of the multi-strain formulations that produced some of the largest CRP reductions in our dataset, particularly in psoriasis. Since reductions in IL-6 and TNF-α occur gradually as barrier integrity improves and innate immune signaling stabilizes, the extent of CRP suppression may depend on how long these pathways remain downregulated. Mice studies also support a time-dependent pattern, with Tsai et al. showing that longer periods of probiotic feeding produce more persistent immune modulation than shorter exposures, suggesting that extended treatment may be necessary for the full downstream anti-inflammatory effects observed in our >8-week CRP subgroup [62]. Further, *Bifidobacterium longum* and *B. breve* potentially inhibit macrophage NF-κB signaling, leading to reduced transcription of IL-6 and TNF-α, which would both induce an increase in CRP from the hepatocytes [63]. For example, *B. longum* was seen in RA and hypothyroidism synbiotic trials, while *B. breve* appeared in psoriasis formulations that produced marked CRP reductions, supporting the idea that Bifidobacteria-driven cytokine suppression translates into measurable systemic effects. VSL#3, a probiotic mix containing *Streptococcus thermophilus*, *Bifidobacteria*, and various *Lactobacillus* strains, was shown to help tighten the gut barrier by reducing claudin-2 levels in the colon [64]. Note that the formula of VSL#3 has been altered since 2016 following a chance in product ownership. Animal models show that multi-strain formulations produce stronger suppression of mucosal IL-6 and TNF-α, cytokines that directly drive hepatic CRP synthesis, resulting in larger downstream reductions in circulating CRP, perhaps explaining our observation of greater CRP improvements in the multi-strain interventions than the single strain [65].

These mechanistic pathways can lead to reduced inflammation in ADs. In mouse models with colitis, a VSL#3, a probiotic mix reduced gut permeability and lowered liver exposure to inflammatory triggers, which in turn led to a drop in serum CRP and mucosal IL-6 and TNF-α levels [64]. In psoriasis, CRP potentially decreased, leading to downregulation of IL-23 and IL-17A, both of which are downstream of TNF-α and IL-6 and are sensitive to microbial modulation of dendritic-cell signaling [66]. In T1DM models, SCFA-producing strains like *Clostridium butyricum* restore barrier function and reduce systemic IL-1β and TNF-α, with non-obese diabetic (NOD) mice showing lower CRP levels when treated with butyrate and acetate [67]. Moreover, GPR43-deficient mice, which lack SCFA sensing, show exaggerated CRP and proinflammatory cytokine responses [68]. Altogether, CRP reduction across these ADs likely reflects convergence on shared inflammatory pathways, namely IL-6, TNF-α, and gut-derived immune activation, that are targeted by microbial therapies.

### 4.2. IL-6

Our meta-analysis did not demonstrate a significant overall reduction in IL-6 and was characterized by extreme heterogeneity (I^2^ = 96%). Across primary and sensitivity analyses, no statistically significant association was observed between probiotic interventions and IL-6 levels. Exclusion of the study assessed as high risk of bias did not materially alter the direction or significance of the pooled estimate, indicating a stable null finding. Taken together, these results suggest that current evidence does not support a robust effect of probiotics on IL-6, and any potential influence should be considered exploratory.

However, the patterns across disease and intervention subgroups could suggest that this effect was only not significant overall due to the heterogeneity. IL-6 is a pleiotropic proinflammatory cytokine central to AD pathogenesis. It is produced primarily by activated macrophages, dendritic cells, and intestinal epithelial cells in response to microbial products like LPS via TLR4–NF-κB signaling. Once secreted, IL-6 activates the gp130-JAK-STAT3 signaling cascade in hepatocytes and immune cells, driving systemic inflammation, acute-phase reactant synthesis, and effector T-cell differentiation [69]. Elevated IL-6 levels in ADs are closely tied to barrier dysfunction and gut-derived immune activation. The variability in overall effect mirrors findings from a meta-analysis of probiotic therapy in critically ill and trauma ICU patients, where IL-6 responses remained highly inconsistent despite clear CRP reductions, suggesting that IL-6 may be a more context-dependent and less reliably modifiable cytokine [70]. Although no significant overall reduction in IL-6 was observed, decreases were seen in certain disease subgroups, suggesting that probiotic effects on IL-6 may depend on how the intervention is delivered rather than reflecting a uniform effect. IL-6 regulation is complex and may require sustained exposure to achieve measurable systemic changes (Figure 7). In this regard, longer intervention durations were associated with greater IL-6 reductions, while shorter courses showed little effect. Differences in probiotic formulations, including strain composition and functional activity, may also influence responsiveness across conditions. These findings suggest that modulation of IL-6 by probiotic interventions is likely context-dependent and may be more evident in diseases with active IL-6 driven inflammation.

Several probiotic strains are hypothesized to decrease the IL-6 expression by affecting these upstream immune pathways, although these effects are not uniform across all settings. *Lactobacillus rhamnosus* reduces LPS translocation by enhancing epithelial tight junctions through EGFR signaling, thereby, in theory, dampening NF-κB activation in mucosal immune cells and reducing IL-6 transcription [61]. In the included psoriasis trials, *L. rhamnosus* was part of multi-strain probiotic formulations that produced some of the largest IL-6 reductions, consistent with its barrier-enhancing and NF-κB suppressive effects, suggesting that barrier-enhancing strains may exert stronger effects in diseases with prominent epithelial dysfunction. LPS translocation by *Bifidobacterium longum* and *B. breve* inhibit IL-6 gene expression in macrophages by suppressing NF-κB activity [63]. As previously mentioned, VSL#3 has been shown to reduce colonic permeability in mouse models by downregulating claudin-2. This indirectly limits microbial antigen access to submucosal immune cells; this was shown to decrease mucosal and serum IL-6 levels, improving histologic inflammation scores [64]. This could be potentially applied to human models. In our dataset, similar multi-strain formulations were used in UC and mixed IBD trials, all of which demonstrated IL-6 reductions. However, these effects were not consistent across all trials or disease categories, reflecting the substantial heterogeneity also observed in our pooled IL-6 estimate.

The variability in the meta-analysis is not unexpected given that IL-6 responses appear to be highly disease-specific and strain-dependent. IL-6 suppression has therapeutic implications across ADs as a disease specific marker. In RA, IL-6 promotes synovial fibroblast proliferation and B-cell activation, both of which are inhibited in germ-free or probiotic-treated models [71]. In psoriasis, IL-6 allows for dendritic cell induced Th17 differentiation, contributing to IL-17A mediated epidermal hyperplasia; microbial therapies reduce this IL-6- IL-23- Th17 axis [66]. SCFA-producing probiotics like *Clostridium butyricum* reduce systemic IL-6 in NOD mice, contributing to β-cell preservation [67]. This parallels the T1DM clinical trial in our dataset, where *C. butyricum* supplementation contributed to IL-6 reductions, matching the known butyrate-mediated signaling effects. Moreover, in UC, reduced IL-6 after probiotic or synbiotic treatment correlates with improved mucosal healing and decreased disease activity.

Taken together, these disease-specific signals suggest that IL-6 responsiveness to microbial therapies may depend heavily on the underlying immunologic architecture of each autoimmune disease, which could explain why meaningful IL-6 reductions emerged in several individual conditions even though the overall pooled effect was not significant. Improving gut barrier function and calming innate immune signals with nutraceuticals can help reduce IL-6 levels at several steps along the inflammatory pathway.

### 4.3. IL-10

Across the studies we analyzed, IL-10 levels rose following probiotic or synbiotic therapy, reflecting enhanced activation of regulatory pathways that counteract IL-6 and TNF-α–mediated inflammation. The significant overall increase in IL-10 across trials suggests that microbial therapies can reliably augment regulatory immune pathways. IL-10 responses were heterogeneous across studies (I^2^ = 98%), despite a positive pooled estimate. Although the primary analysis suggested an increase in IL-10 levels following probiotic interventions, this association was no longer statistically significant after exclusion of the study assessed as high risk of bias. This sensitivity to study quality indicates that the observed effect on IL-10 is not robust and may be driven, at least in part, by lower-quality evidence. Accordingly, findings related to IL-10 should be interpreted cautiously and considered exploratory rather than confirmatory

IL-10 is a central anti-inflammatory mediator that helps keep immune responses in check, especially along mucosal surfaces. It is mainly released by regulatory T cells, macrophages, and dendritic cells in response to microbial products or ongoing inflammation [72]. IL-10 works by dampening key inflammatory pathways like NF-κB and Mitogen-activated protein kinase (MAPK), leading to reduced expression of cytokines such as IL-6, TNF-α, and IL-1β. It also limits the activity of antigen-presenting cells by suppressing costimulatory signals and Major histocompatibility complex (MHC) expression, helping to prevent excessive immune activation [72]. In ADs, IL-10 dysregulation is associated with exacerbated inflammation and tissue damage. Gut-derived inflammation, particularly in conditions with increased epithelial permeability, could reduce IL-10 signaling and shift the immune balance toward a pro-inflammatory state. The consistent direction of effect despite marked heterogeneity shows that IL-10 upregulation may represent a shared downstream response across multiple probiotic formulations.

Certain probiotics and synbiotics can raise IL-10 levels by supporting the development of immune cells that limit inflammation. The absence of a clear difference between single and multi-strain products suggests that IL-10 responses may be driven more by the specific immunologic effects of certain strains rather than by the number of strains included. In our analysis, single-strain interventions consistently increased IL-10. This pattern aligns with experimental work showing that species such as *L. reuteri*, *B. infantis*, and *C. butyricum* can directly promote IL-10 production through dendritic-cell signaling and expansion of Foxp3+ regulatory T cells. Multi-strain formulations showed a larger but more inconsistent effect, which may reflect differences in strains, dosing, and how the individual strains interact once delivered together. *Lactobacillus plantarum* and *Bifidobacterium infantis* have been shown to increase IL-10 mRNA expression in mice with Crohn’s disease [73]. *Lactobacillus reuteri* restored IL-10 expression in the intestines of LPS-challenged mice, implying that part of its anti-inflammatory effect may come from re-engaging the body’s own IL-10-mediated control of inflammation [74]. This data could theoretically explain our meta-analysis, where *L. plantarum* and *B. infantis* appeared in several IBD-focused probiotic combinations, particularly in UC. This showed IL-10 increases alongside reductions in pro-inflammatory cytokines. Further, another formulation, *Clostridium butyricum*, enhances IL-10 by promoting the expansion of regulatory T cells in the colon, likely through histone acetylation [75]. These mechanisms likely underlie the observed IL-10 increases in animal models of autoimmune inflammation. In colitis mice, synbiotic supplementation with *Bifidobacterium longum* elevated colonic and systemic IL-10, which correlated with decreased mucosal TNF-α and IL-6 and histologic healing [76]. In NOD mice, SCFA-producing probiotic strains such as *Clostridium* clusters increased systemic IL-10 and protected against β-cell autoimmunity [67].

In our meta-analysis, shorter interventions (≤8 weeks) showed clearer IL-10 increases, whereas longer courses had more variable effects. This pattern is consistent with IL-10 working as an early regulatory response to microbial signaling, particularly through TLR2 dendritic cell pathways, rather than a marker that steadily rises with continued exposure. An animal study supports this early-phase idea; in NOD mice, probiotic administration rapidly induced IL-10 production [77]. Whether this response plateaus or declines with an extended treatment is uncertain, but the available evidence suggests that IL-10 kinetics differ from markers such as CRP or IL-6, which generally require longer intervention periods to show maximal change. This matches the pattern seen in the T1DM trial in our dataset, which used SCFA-producing strains, *Lactobacillus salivarius* subsp. *salicinius AP-32*, *Lactobacillus johnsonii MH-68*, and *Bifidobacterium animalis* subspecies, and demonstrated high IL-10 elevation, consistent with restoration of mucosal immune tolerance. Further, this idea of early phase effect may be why low dose improved IL-10 more than high dose, as a high dose may tip the balance away from Treg-mediated IL-10 production and toward pro-inflammatory responses [75].

The increase in IL-10 after microbial supplementation likely signals a recovery of mucosal and reduction in inflammation. In ADs, this helps keep IL-6 and TNF-α in check and supports a more balanced immune response.

### 4.4. TNF-α

TNF-α levels fell across multiple trials, pointing to a shared ability of these interventions to quiet one of the primary drivers of systemic inflammatory activity. As with CRP, the observed reduction in TNF-α occurred in the context of extreme heterogeneity (I^2^ = 96%), suggesting that the magnitude and consistency of effect varied markedly between studies. In contrast to IL-6 and IL-10, however, the association between probiotic interventions and TNF-α levels remained statistically significant after exclusion of the study assessed as high risk of bias, with minimal residual heterogeneity. This robustness across sensitivity analyses suggests that the observed reduction in TNF-α is less likely to be driven by methodological limitations of individual studies and may represent a more consistent immunomodulatory effect. TNF-α is a key upstream proinflammatory cytokine that drives chronic inflammation in ADs. It is secreted mainly by activated macrophages, dendritic cells, and T cells in response to microbial products such as LPS, which activate the TLR4-MyD88-NF-κB pathway [78]. TNF-α then promotes the transcription of other inflammatory mediators including IL-6 and IL-1β, amplifies antigen presentation by upregulating MHC class II on antigen-presenting cells, and brings additional immune cells through chemokine processes [78].

Intervention type was the only factor that significantly shaped TNF-α outcomes. Multi-strain probiotic formulations were associated with larger reductions in TNF-α compared with single-strain preparations or fecal microbiota transplantation, which may reflect broader functional activity across multiple bacterial strains. *Bifidobacterium longum* and *Lactobacillus casei* could suppress NF-κB activation in intestinal macrophages, reducing TNF-α gene transcription [79]. Both strains were also used across several autoimmune-focused trials in our dataset, including RA, UC, and SLE, where formulations containing *B. longum* or *L. casei* demonstrated measurable reductions in TNF-α. In contrast, treatment duration and dose did not significantly modify TNF-α responses. Dose-stratified analyses showed significant reductions at both low and high doses, without evidence of a clear dose–response relationship, suggesting that TNF-α suppression may occur within a threshold range rather than increasing proportionally with higher doses. All together, these trends suggest that TNF-α suppression is driven primarily by the composition and microbiota diversity of the intervention, rather than the dose or duration of exposure.

Suppressing TNF-α has demonstrated potential benefits across ADs. In RA, TNF-α pushes the synovial inflammation and joint destruction through activation of fibroblast-like synoviocytes. Several small clinical studies have shown that probiotic-induced reductions in serum TNF-α correlate with decreases in CRP levels [80,81]. In UC, excess TNF-α disrupts epithelial integrity and promotes neutrophil infiltration; interestingly, rodent models treated with synbiotics such as *B. longum* exhibit lower mucosal TNF-α and improved barrier function [76]. This corresponds to the UC synbiotic trials in our dataset, which also used *B. longum*-containing formulations and reported reductions in TNF-α alongside improved inflammation scores. In T1DM, TNF-α contributes to β-cell destruction and promotes the activation of autoreactive T cells. As an intervention, SCFA-producing probiotics have been shown to lower TNF-α levels and help preserve the crucial β-cell mass in NOD mice. [67]. By dampening inflammation along the gut-liver axis, microbial modulation of TNF-α may help lower overall systemic inflammation. [66].

Taken together, the evidence suggests that TNF-α serves as a driver of inflammation across autoimmune diseases. By modulating gut-immune interactions, microbial therapies may help quiet this inflammatory pathway and re-establish systemic balance.

### 4.5. Additional Inflammatory Markers

Beyond core cytokines such as IL-6, TNF-α, and IL-10, there are other markers that had modest evidence, showing benefits following the use of these interventions. These include the epithelial chemokine IL-8, the Th17/IL-23 axis, the inflammasome IL-1β pathway, and redox-sensitive mediators like NO and TAC.

IL-8 is a neutrophil chemoattractant secreted by intestinal epithelial and stromal cells in response to microbial-associated molecular patterns (MAMPs) via TLR NF-κB activation. Elevated IL-8 drives mucosal injury and neutrophilic infiltration in UC and T1DM. Multi-strain probiotics reduced circulating IL-8 in both diseases, likely via restoration of barrier function and downregulation of TLR4 signaling, limiting epithelial-derived cytokine release [82,83]. These IL-8 reductions were most apparent in trials using *Lactobacillus* and *Bifidobacterium* based combinations, the same multi-strain formulations used across UC, MS, and psoriasis studies in our dataset, several of which reported directionally similar decreases in epithelial-derived inflammatory markers.

IL-23 and IL-17, central to Th17 cell survival and tissue infiltration, are amplified by the dysbiosis of microbiota through dendritic cell activation and excessive IL-6/IL-1β signaling. In UC, FMT significantly lowered both IL-17 and IL-23 levels at an effect similar to what is seen with glucocorticoids, often a standard treatment. It was further suggested that it helped restore gut-immune balance by reducing dendritic cell IL-23 production [82]. Interestingly, IL-17 levels rose slightly in AS following synbiotic therapy, which may reflect ongoing mucosal immune activity or a compensatory shift in the immune response [44]. Notably, the AxSp synbiotic study in our dataset used *B. longum* with inulin, a formulation that increased multiple immune mediators in previous trials and may similarly have contributed to this modest rise in IL-17. Rather than bluntly suppressing Th17 activity, microbial therapies may help fine-tune it in a disease-specific way.

Finally, NO and TAC reflect the body’s overall ability to neutralize oxidative stress. Increased TAC observed in MS patients following high-dose probiotics may result from SCFA-mediated activation of the Nrf 2 pathway, which upregulates glutathione, catalase, and superoxide dismutase expression thereby enhancing mucosal antioxidant defenses and limiting reactive oxygen species-induced tissue injury [39,84]. The MS trials in our dataset used multi-strain mixtures containing *Lactobacillus*, *Bifidobacterium*, and *Streptococcus thermophilus*, several of which have documented SCFA-enhancing or antioxidant-modulating properties consistent with the TAC improvements reported.

## 5. Limitations

The evidence base for probiotics, synbiotics, and FMT in autoimmune diseases remains limited in size and duration. Most trials enrolled fewer than 50 participants per arm and lasted no longer than 12 weeks. This makes them underpowered to reliably detect effects such as relapse prevention, disease progression, or organ protection. The heterogeneity of interventions adds further difficulty: studies varied in strain composition (single vs. multi-species), dosing (10^6^–10^11^ CFU/day), inclusion of prebiotics, and delivery route (capsule, sachet, enema, FMT via multiple approaches). Outcomes were also inconsistent, with trials emphasizing biomarkers (CRP, IL-6, TNF-α, IL-17) over standardized clinical indices, and disease activity measures differing even within the same condition. Background medications (DMARDs, mesalamine, interferons, steroids) were nearly universal, obscuring the independent effect of the nutraceutical. Few studies accounted for host factors such as baseline microbiome composition, dietary fiber intake, or disease phenotype, and very few included microbiome sequencing or metabolomics to verify mechanistic pathways.

The strongest evidence comes from UC, though most trials were short-term and rarely tested relapse prevention. FMT studies also varied in donor selection, preparation, and dosing, limiting comparability. For CD, evidence is very limited; aside from one larger trial with *Saccharomyces boulardii*, most studies were small and combined probiotics with mesalamine. In MS, results are encouraging but limited by heterogeneity in patient subtypes, background therapy, and short follow-up, meaning the evidence on how it affects relapse and disability is uncertain.

For less-studied conditions such as SLE, T1DM, psoriasis, ERA-JIA, axial spondylarthritis, and hypothyroidism, the evidence comes from only a handful of small trials. The studies suggest some biomarker shifts but limited or inconsistent clinical benefit, with hypothyroidism even showing a paradoxical CRP increase. This limits our capability to draw any conclusions about the intervention applicability to these specific diseases.

Further, findings of this meta-analysis should be interpreted in light of several limitations. Although pooled analyses demonstrated statistically significant effects for CRP, TNF-α, and IL-10, these findings were accompanied by substantial between-study heterogeneity, with I^2^ values exceeding 80% for all significant outcomes. Although sensitivity analyses excluding high risk-of-bias studies reduced heterogeneity for some outcomes, residual heterogeneity persisted in several analyses. This suggests that unmeasured clinical and methodological differences such as baseline disease activity, background immunosuppressive therapy, and microbiome assessment methods may have influenced effect estimates. Further, publication bias could only be formally assessed for CRP due to an adequate number of studies. For other cytokines, the limited number of available studies precluded meaningful funnel plot–based analyses, and the potential influence of publication bias on these outcomes cannot be excluded.

## 6. Future Perspectives

Future research on gut-targeted nutraceuticals in ADs needs to move beyond small pilot studies and towards a precision medicine approach with large clinical trials. Current trials show that these interventions can lower CRP, IL-6, TNF-α, and other inflammatory cytokines, but turning these effects into lasting clinical benefit will require filling several important gaps.

First, formulations need to be standardized. Right now, the field is scattered across different strain mixes, doses, and delivery methods, which makes it hard to compare results or build on past work. Developing a reference product, such as a generalized, core *Lacto-Bifido* synbiotic, would let future trials generate cumulative evidence instead of one-off findings. At the same time, treatments should be tailored to each disease. In RA, for example, strains with antigen-modulating properties like exopolysaccharide-producing Lactobacillus may be more effective, while in UC and MS, multi-strain blends paired with fermentable fiber could work best by boosting Tregs and repairing barrier function.

Second, trials should track mechanisms as well as symptoms. Looking at the microbiome itself, SCFA and bile acids, gut barrier markers, and Treg/Th17 balance can show if the treatment is working accurately, rather than just causing general changes. Using broader tools like metagenomics, metabolomics, and immune profiling can also help pick out who responds best to which intervention, so future studies can target the right patients.

Third, studies need to take host and disease context into account. Factors like baseline microbiota, fiber intake, genetics, and concurrent immunotherapy all influence response but are rarely measured. Smarter trial designs could identify subgroups that benefit most, like CD patients lacking butyrate producers or MS patients with a strong Th17 profile.

The integration of artificial intelligence (AI) and machine learning (ML) algorithms may greatly accelerate progress and improve outcomes. Their use in the analysis of multi-omics data including microbiome sequencing, metabolomics, genomics, proteomics, and immunophenotyping can reveal complex interactions that our current methodologies overlook. Moreover, predictive and adaptive models could identify biomarker signatures that forecast which patients will respond to specific nutraceutical formulations and optimize strain selection and dosing in real time. For instance, RA cohorts with distinct Th17/IL-23 axis activity or UC patients with specific SCFA profiles could be algorithmically matched to synbiotic combinations most likely to induce regulatory T cell expansion or epithelial repair.

Finally, trial design needs to evolve. Most studies to date are short (≤12 weeks), underpowered, and use biomarkers as primary outcomes. Future RCTs should be longitudinal (≥6–12 months), powered, and designed to test clinical endpoints: relapse prevention in IBD, DAS-28 or radiographic progression in RA, relapse rate and EDSS in MS, nephritis control in SLE, or HbA1c and C-peptide preservation in T1DM. Combination strategies should also be explored, pairing nutraceuticals with diet, immunotherapy, or biologics may achieve synergy, particularly in CD and RA where standalone microbiome modulation has been insufficient.

## 7. Conclusions

Gut-modulating therapeutics hold promise in reducing inflammation in ADs. This meta analysis shows that gut-targeted therapies like probiotics, synbiotics, and FMT can help regulate inflammation in ADs by reducing key markers like TNF-α and CRP, and boosting IL-10, which supports immune balance, as well as many others. While the overall results are encouraging, the findings should be interpreted cautiously due to existing limitations in study design, patients, and methodology. Substantial to extreme heterogeneity was observed across outcomes (I^2^ ranging from 80% to 98%), reflecting considerable clinical, methodological, and even disease-related variability among included studies. Nevertheless, this review provided invaluable insights into the existing literature, providing information on the status of research. Future studies should focus on having a larger and more diverse population with standardized methodologies to provide accurate and robust evidence of the benefit of these readily available nutraceuticals. ADs still lack definitive treatment strategies; gut microbiome-modulating therapies, such as probiotics, synbiotics, and FMT may serve as adjunctive therapeutic options. Encouraging their integration into more robust clinical trials could bring positive biomarker alterations, while paving the way for more personalized treatment approaches.

## Data Availability

No new data was created in this study. The data that support the findings of this study are included within the article and Supplementary Materials.

## Supplementary Materials

The following supporting information can be downloaded at: https://www.mdpi.com/article/10.3390/nu18040560/s1, Figure S1: Funnel plot for assessment of publication bias in the meta-analysis of probiotic, prebiotic, and synbiotic interventions on CRP levels. Each circle represents an individual study plotted by standardized mean difference (x-axis) against standard error (y-axis). The vertical dashed line indicates the pooled effect estimate from the random-effects model (SMD = −0.67). Shaded regions represent the 90% (light gray), 95% (medium gray), and 99% (dark gray) confidence interval contours. A symmetric distribution around the pooled estimate suggests low risk of publication bias.; Figure S2: Forest plots of SMDs in CRP levels across probiotic intervention studies, stratified by (A) intervention type, (B) intervention duration, (C) dose category, and (D) underlying disease. Boxes represent individual study effect sizes with 95% confidence intervals; diamonds indicate pooled random-effects estimates and corresponding 95% confidence intervals for each subgroup and overall.; Figure S3: Forest plots of SMDs in IL-6 levels across probiotic intervention studies, stratified by (A) intervention type, (B) intervention duration, (C) dose category, and (D) underlying disease. Boxes represent individual study effect sizes with 95% confidence intervals; diamonds indicate pooled random-effects estimates and corresponding 95% confidence intervals for each subgroup and overall.; Figure S4: Forest plots of SMDs in IL-10 levels across probiotic intervention studies, stratified by (A) intervention type, (B) intervention duration, (C) dose category, and (D) underlying disease. Boxes represent individual study effect sizes with 95% confidence intervals; diamonds indicate pooled random-effects estimates and corresponding 95% confidence intervals for each subgroup and overall.; Figure S5: Forest plots of SMDs in TNF-α levels across probiotic intervention studies, stratified by (A) intervention type, (B) intervention duration, (C) dose category, and (D) underlying disease. Boxes represent individual study effect sizes with 95% confidence intervals; diamonds indicate pooled random-effects estimates and corresponding 95% confidence intervals for each subgroup and overall.

## Abbreviations

ADs	Autoimmune diseases
AI	Artificial intelligence
BMI	Body mass index
CD	Crohn’s disease
CFU	Colony forming unit
CRP	C-reactive protein
DCs	Dendritic cells
DMARDs	Disease-modifying antirheumatic drugs
EGFR	Epidermal growth factor receptor
ESR	Erythrocyte sedimentation rate
FMT	Fecal microbiota transplantation
GSH	Glutathione
HbA1c	Hemoglobin A1c
IBD	Inflammatory bowel disease
IL-1β	Interleukin-1 beta
IL-6	Interleukin-6
IL-8	Interleukin-8
IL-10	Interleukin-10
IL-12	Interleukin-12
IL-17	Interleukin-17
IL-23	Interleukin-23
IQR	Interquartile range
LPS	Lipopolysaccharide
MAPK	Mitogen-activated protein kinase
MAMPs	Microbial-associated molecular patterns
MDA	Malondialdehyde
MHC	Major histocompatibility complex
ML	Machine learning
MS	Multiple sclerosis
NF-κB	Nuclear factor kappa-light-chain-enhancer of activated B cells
NOD	Non-obese diabetic (mouse model)
NO	Nitric oxide
PBC	Primary biliary cholangitis
PRISMA	Preferred Reporting Items for Systematic Reviews and Meta-Analyses
RA	Rheumatoid arthritis
ROS	Reactive oxygen species
SCFA	Short-chain fatty acid
SLE	Systemic lupus erythematosus
SMD	Standardized mean difference
TAC	Total antioxidant capacity
T1DM	Type 1 diabetes mellitus
TGF-β	Transforming growth factor-beta
TLR	Toll-like receptor
TNF-α	Tumor necrosis factor-alpha
UC	Ulcerative colitis
