# Supplementary material for: The Effects of Microbiome Modulating Therapies on Inflammatory Markers in Autoimmune Disease: A Systematic Review and Meta-Analysis
Source: Nutrients. 2026 Feb 8;18(4):560. doi: 10.3390/nu18040560 (PMC12943447; doi:10.3390/nu18040560)
Supplement: Supplementary file 1 [file nutrients-18-00560-s001.zip › nutrients-4067174-supplementary.pdf]

**Supplementary Material**

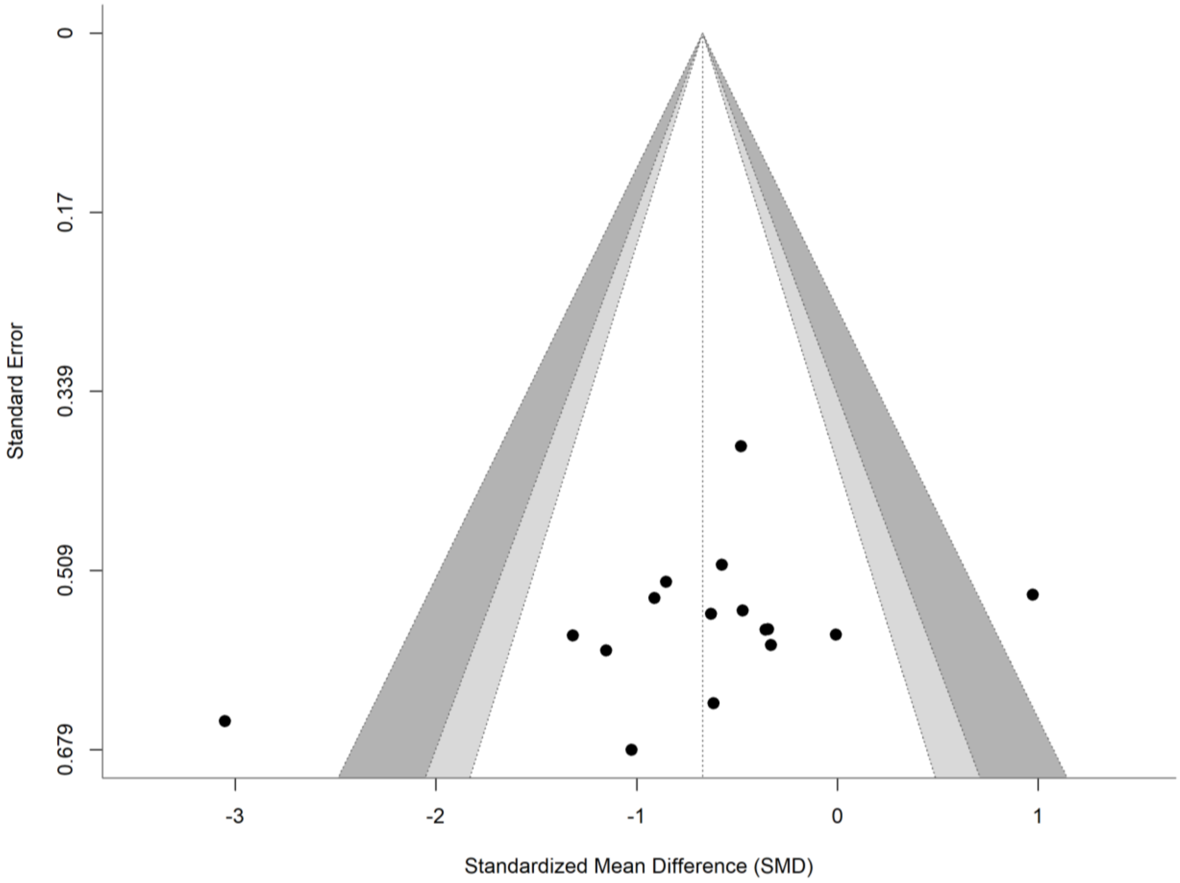


**Supplementary Figure 1:** Funnel plot for assessment of publication bias in the meta-analysis of probiotic, prebiotic, and synbiotic interventions on CRP levels. Each circle represents an individual study plotted by standardized mean difference (x-axis) against standard error (y-axis). The vertical dashed line indicates the pooled effect estimate from the random-effects model (SMD = -0.67). Shaded regions represent the 90% (light gray), 95% (medium gray), and 99% (dark gray) confidence interval contours. A symmetric distribution around the pooled estimate suggests low risk of publication bias.

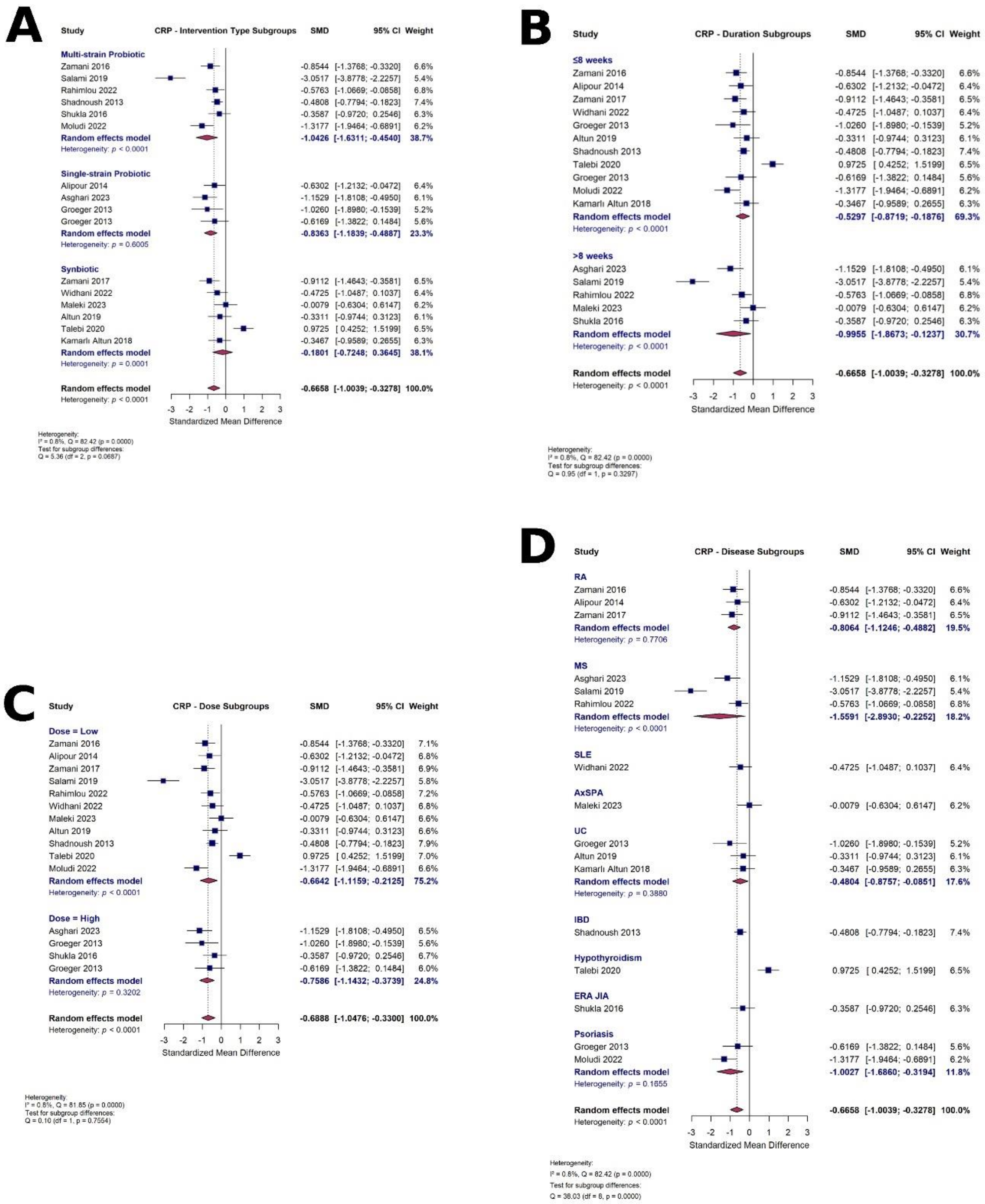

**Supplementary Figure 2:** Forest plots of SMDs in CRP levels across probiotic intervention studies, stratified by (A) intervention type, (B) intervention duration, (C) dose category, and (D) underlying disease. Boxes represent individual study effect sizes with 95% confidence intervals; diamonds indicate pooled random-effects estimates and corresponding 95% confidence intervals for each subgroup and overall.

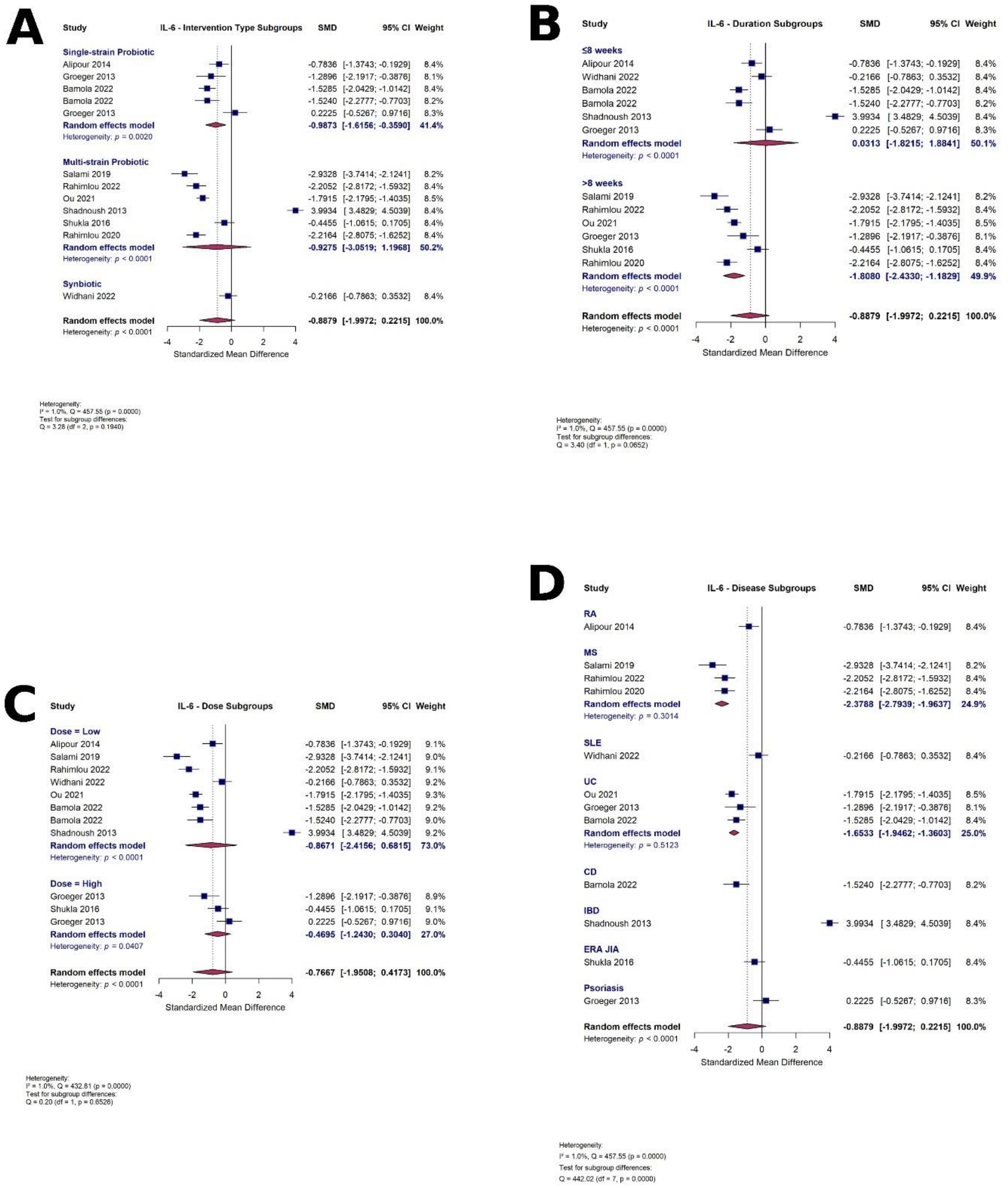


**Supplementary Figure 3:** Forest plots of SMDs in IL-6 levels across probiotic intervention studies, stratified by (A) intervention type, (B) intervention duration, (C) dose category, and (D) underlying disease. Boxes represent individual study effect sizes with 95% confidence intervals; diamonds indicate pooled random-effects estimates and corresponding 95% confidence intervals for each subgroup and overall.

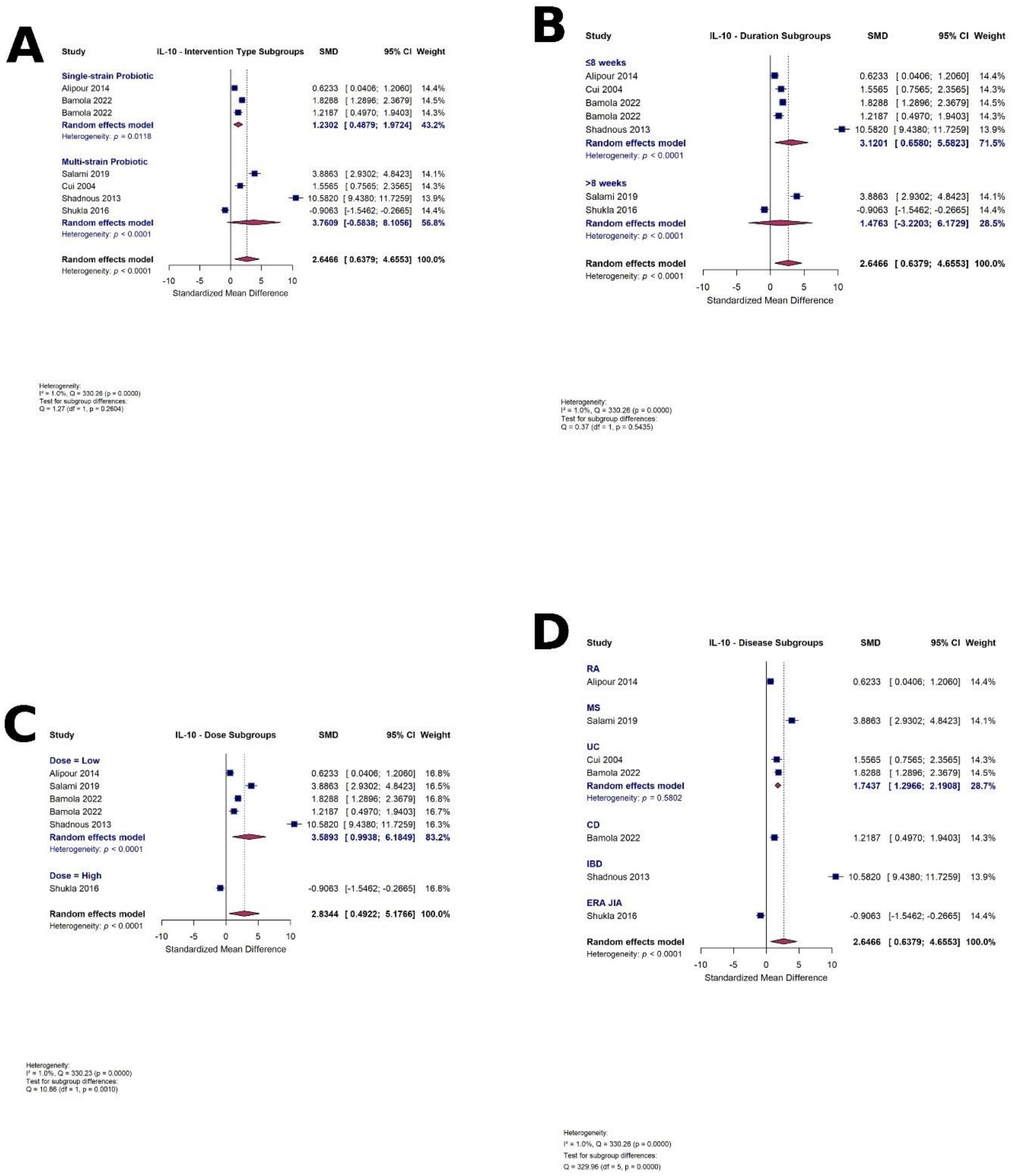


**Supplementary Figure 4:** Forest plots of SMDs in IL-10 levels across probiotic intervention studies, stratified by (A) intervention type, (B) intervention duration, (C) dose category, and (D) underlying disease. Boxes represent individual study effect sizes with 95% confidence intervals; diamonds indicate pooled random-effects estimates and corresponding 95% confidence intervals for each subgroup and overall.

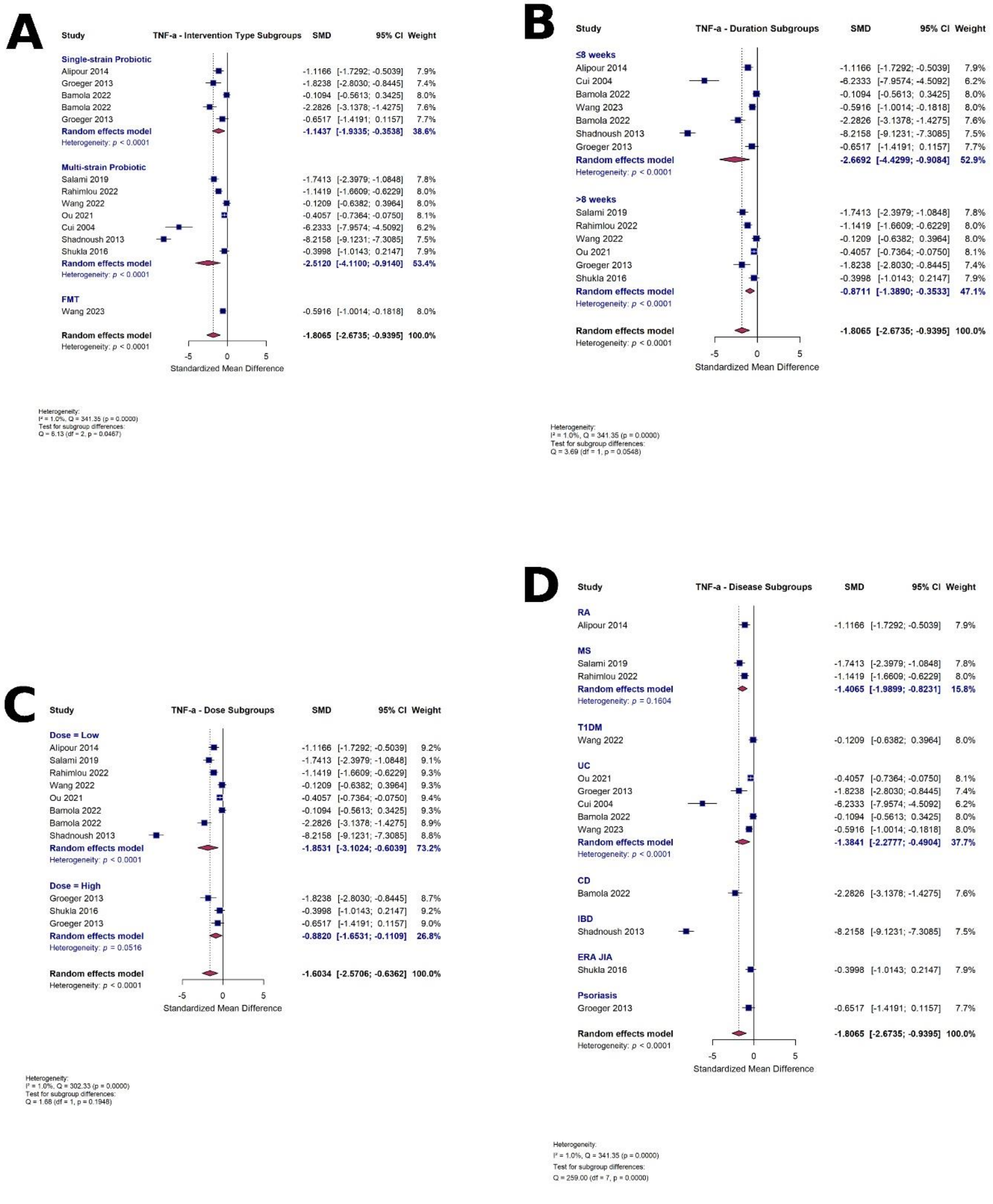


**Supplementary Figure 5:** Forest plots of SMDs in TNF-$\alpha$ levels across probiotic intervention studies, stratified by (A) intervention type, (B) intervention duration, (C) dose category, and (D) underlying disease. Boxes represent individual study effect sizes with 95% confidence intervals; diamonds indicate pooled random-effects estimates and corresponding 95% confidence intervals for each subgroup and overall.
